# Primate DNA suggests long-term stability of an African rainforest

**DOI:** 10.1002/ece3.395

**Published:** 2012-10-09

**Authors:** Julie M Allen, Michael M Miyamoto, Chieh-Hsi Wu, Tamar E Carter, Judit Ungvari-Martin, Kristin Magrini, Colin A Chapman

**Affiliations:** 1 Florida Museum of Natural History, Dickinson Hall, Museum Road and Newell Drive, University of FloridaGainesville, Florida, 32611; 2 Department of Biology, University of FloridaBox 118525, Gainesville, Florida, 32611-8525; 3 The Bioinformatics Institute, University of AucklandPrivate Bag, Auckland, 92019, New Zealand; 4 Genetics Institute, University of FloridaGainesville, Florida, 32611-8525; 5 Department of Anthropology & McGill School of Environment, McGill UniversityMontreal, Quebec, H3A 2T7, Canada; 6 Wildlife Conservation Society185th Street and Southern Boulevard, Bronx, New York, 10460

**Keywords:** Coalescent theory, conservation biology, historical demography, microsatellites, red colobus

## Abstract

Red colobus monkeys, due to their sensitivity to environmental change, are indicator species of the overall health of their tropical rainforest habitats. As a result of habitat loss and overhunting, they are among the most endangered primates in the world, with very few viable populations remaining. Traditionally, extant indicator species have been used to signify the conditions of their current habitats, but they have also been employed to track past environmental conditions by detecting previous population fluctuations. Kibale National Park (KNP) in Uganda harbors the only remaining unthreatened large population of red colobus. We used microsatellite DNA to evaluate the historical demography of these red colobus and, therefore, the long-term stability of their habitat. We find that the red colobus population throughout KNP has been stable for at least ∼40,000 years. We interpret this result as evidence of long-term forest stability because a change in the available habitat or population movement would have elicited a corresponding change in population size. We conclude that the forest of what is now Kibale National Park may have served as a Late Pleistocene refuge for many East African species.

## Introduction

During the Pleistocene and into the Holocene, there have been large faunal and floral changes due both to anthropogenic factors and climate change (Gill et al. 2009; Nogués-Bravo et al. 2010; McGlone 2012; Prescott et al. 2012; Rule et al. 2012). Understanding the magnitude of each of these issues has been incredibly challenging. One method to detect environmental change is to determine when species populations have fluctuated (Goossens et al. 2006; Schneider et al. 2010; deBruyn et al. 2011; Ting et al. 2012). Although species may respond differently to environmental changes (Arenas et al. 2012; Lorenzen et al. 2011), indicator species are particularly sensitive to minor changes in environmental condition (Lindenmayer et al. 2000), and in many cases, it is understood how they will respond to environmental changes. In fact, these species have been important for monitoring the status of our natural environment and even the presence of other species. Although, traditionally, extant indicator species have been used to indicate the conditions of their current environment, by looking at past population demographics (e.g., population bottlenecks or expansions), they may also be used to indicate the history of their habitat.

Red colobus monkeys, genus *Procolobus[Piliocolobus]* (Oates and Davies 1994; Grubb et al. 2003), are indicator species (Chapman and Lambert 2000; Waltert et al. 2002; Struhsaker 2005, 2010) because they are sensitive to habitat change (Oates and Davies 1994; Waltert et al. 2002). Their populations are thought to signify the overall condition of their tropical rainforest. With rapid habitat loss across equatorial Africa and hunting in many areas, they are considered one of the most endangered primates in the world (Struhsaker 2005; Mittermeier et al. 2009). Although the taxonomy of red colobus has been under considerable debate for some time, there are 18 morphotypes currently recognized across equatorial Africa (Grubb et al. 2003; Ting 2008). In 2008, 13 morphotypes were evaluated by the IUCN and all but one was in decline and many were critically endangered. Unfortunately, little is known of the natural history of many of these morphotypes and the majority of red colobus habitat remains unprotected making the future of this genus very questionable (Struhsaker 2005).

**Figure 1:**
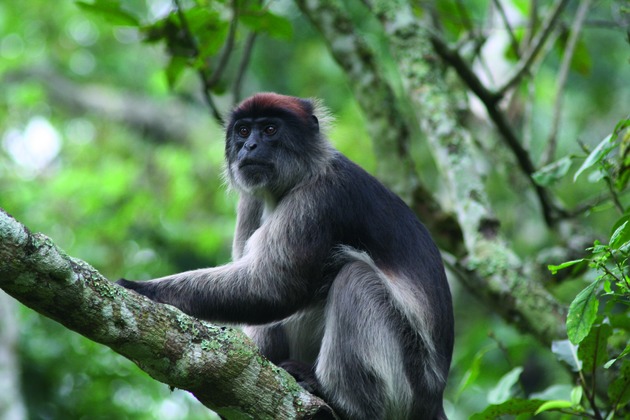
Red colobus monkey, *Procolobus [Piliocolobus] rufomitratus*, from Kibale National Park, Uganda.

*Procolobus [Piliocolobus] rufomitratus* is found in Kibale National Park (KNP) in Western Uganda (Fig. 1), and is one of the few large groups from the entire distribution of this genus that is presently not threatened. The estimated census size (*N*_*c*_) of the red colobus in KNP is ∼17,000 individuals (Struhsaker 2005), which is indicative of a healthy tropical rainforest that occupies a relatively small area of ∼795 km^2^. Kibale National Park supports an extremely high level of biological diversity with more than 351 species of trees, 335 species of birds, and an unprecedented 13 species of primates (Uganda Wildlife Authority 2011). Although there has been much deforestation in Uganda (Howard et al. 2000), KNP has remained relatively stable since early monitoring began in the 1970s (Struhsaker 1975; Southworth et al. 2010; Naughton-Treves et al. 2011). This upland tropical rainforest was designated a national park in 1993 to preserve its biological diversity. The park is a major destination for eco-tourists and has been at the forefront of research on African forest primates for over 40 years (Chapman et al. 2005, 2010a,b; Box et al. 2008; Struhsaker 2010). However, we have little information about the history of Kibale forest, particularly during the glacial and interglacial periods of the Late Pleistocene and into the early Holocene, where we know surrounding areas were affected (Scholz et al. 2007; Stager and Johnson 2008). Similarly, little is known about anthropogenic affects of the forest, apart from the arrival of the Bantu-speaking people around ∼2,300 years ago who are thought to have caused major changes in the forest habitat surrounding Kibale forest (Taylor et al. 1999). As red colobus are very sensitive to habitat change, evidence of past population size changes would likely indicate changes in Kibale forest and shed light on past factors that affected this area.

Microsatellites (DNA regions of short tandem repeats) provide a rich source of polymorphic nuclear DNA markers for studying the genetic structure and diversity of a population's gene pool (Jarne and Lagoda 1996; Cavalli-Sforza 1998; Ellegren 2004). Microsatellite data can be used to look for evidence of past population decline or growth by applying methods based on the coalescent theory and can provide unique insights into the long-term history of a population. Currently, it is not known if all of the red colobus in KNP form one large population made up of many interbreeding groups or if there are different populations within the park. This information gained from microsatellite data for the red colobus in KNP can be compared to other groups of red colobus that are currently declining.

We used microsatellites from six groups of red colobus living throughout KNP to first estimate the number of populations in the park using three different analytical approaches, including Bayesian model testing. We then examined the KNP population for evidence of historical fluctuations in effective population size (*N*_*e*_) using three complementary approaches that included Extended Bayesian Skyline Plots (EBSP) (Heled and Drummond 2008). These models ranged from the familiar stepwise mutation model (SMM) to more complex ones that account for various properties of microsatellite mutation (Sainudiin et al. 2004). Finally, we integrated our population genetic results with field and paleoenvironmental data to infer the history of the forest of Kibale National Park during the Late Pleistocene through the Holocene.

## Materials and Methods

### Sample collection

**Figure 2:**
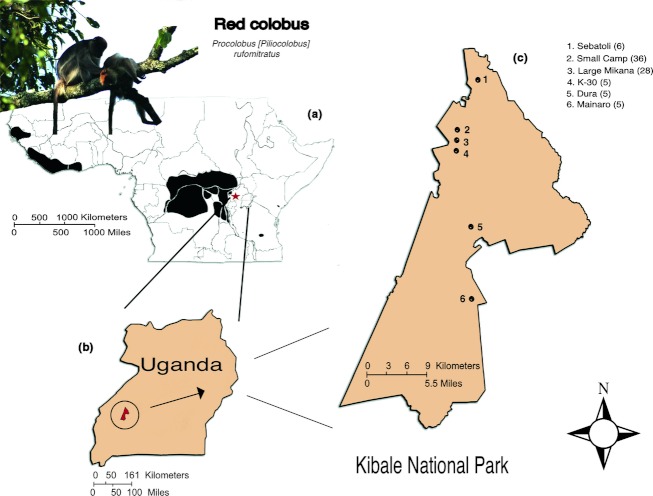
(*a*) Distribution of red colobus across north central Africa (black shading; Ting 2008), with the red star highlighting Kibale National Park (KNP). (*b*) Uganda with the location of KNP in red. (*c*) KNP and the locations of the six groups on the days of their sampling along with their sample sizes (modified from a map by A. Jacob).

Blood and/or fecal material was obtained from 64 individuals of the Large Mikana and Small Camp groups, which are both part of a long-term field study that was started in 2003. Two to four fecal samples were collected from each individual that was identified by distinguishing characteristics (e.g., scars, broken tails, hair color, and birthmarks). In an effort to sample from throughout the park, fecal specimens were also collected from 21 individuals from the Dura (*N* = 5), K30 (*N* = 5), Mainaro (*N* = 5), and Sebatoli (*N* = 6) groups (Fig. 2). The blood samples were stored on classic FTA cards, and the fecal specimens were treated with the two-step procedure described in Nsubuga et al. (2004). Total genomic DNA was extracted from the blood and fecal specimens with the FTA protocol and Qiagen QIAmp®Stool Kit (cat: 51504) according to their manufacturers' recommendations. To obtain adequate DNA for genotyping, DNA was extracted from two to four fecal specimens per individual.

Eleven microsatellite loci from the human genome, found to be polymorphic in red colobus (A.B. Di Fiore, pers. comm.), were selected for genotyping (Table 1). These loci were amplified with a three-primer nested PCR approach (Schuelke 2000), run on an AB3730xl automated sequencer (96 capillary), and scored with GeneMarker® (Softgenetics, State College, PA). All loci were amplified multiple times from multiple extracts per individual to ensure accuracy of genotypes. An earlier version of the red colobus dataset was used by Wu and Drummond (2011) to test the performance of their EBSP models for microsatellites. This earlier dataset included only 31 of the 85 red colobus, which are now represented in the current matrix. Our present EBSP results supersede these previous ones as they are based on all 85 individuals and on direct comprehensive tests for the historical demography of red colobus in KNP (Appendix A1).

**Table 1:** Standard statistics for the 10 microsatellite loci of the 85 red colobus (*Procolobus* [*Piliocolobus] rufomitratus*) from Kibale National Park in Uganda

Locus^a^	*N*^b^	*n*_*a*_	*n*_*e*_	*H*_*o*_	*H*_*e*_	*F*_*IS*_^e^
*D14S306*	84	6	4.25	0.76	0.77	0.01
*D3S1766*	82	9	6.31	0.76	0.85	0.11
*D2S1399*	84	10	6.57	0.91	0.85	−0.06
*D7S1817*	78	9	4.88	0.69	0.8	0.14
*D20S206*^c^	79	7	5.08	0.79	0.81	0.03
*D8S60*	82	4	1.91	0.54	0.48	−0.12
*D8S165*	85	3	2.07	0.55	0.52	−0.06
*D1S207*	85	14	6.12	0.74	0.84	0.12
*C2A*	85	7	2.39	0.55	0.59	0.06
*D561457*	82	8	3.62	0.67	0.73	0.08
**Average**^d^	**82.6**	**7.70**	**4.32**	**0.70**	**0.72**	**0.036**
	(78–85)	(3–14)	(1.91–6.57)	(0.54–0.91)	(0.48–0.85)	(−0.017–0.082)

a Locus nomenclature follows that of the human genome.

b Standard statistics include: (*N*): number of genotyped individuals per locus, (*n*_*a*_) observed number of alleles, (*n*_*e*_) effective number of alleles, (*H*_*o*_) observed heterozygosity, (*H*_*e*_) expected heterozygosity, and (*F*_*IS*_) Wright's *F*_*IS*_.

c This locus is the only one that deviates significantly from Hardy–Weinberg expectations after Bonferroni correction.

d The average for *F*_*IS*_ corresponds to its weighted mean and 95% bootstrap confidence interval across loci. The averages for the other statistics refer to their arithmetic means and ranges.

e *F*_*IS*_ estimates (means and 95% confidence intervals, in parentheses) are 0.10 (0.02–0.17) for Large Mikana and −0.01 (−0.07–0.04) for Small Camp. Thus, total *F*_*IS*_ is not significantly negative for either group with a sample size of >6 individuals. A significantly negative *F*_*IS*_ is indicative of a heterozygote excess and may be related to female philopatry (Chesser 1991). However, dispersal in red colobus is female biased (Struhsaker 2010) and so these results are not unexpected. Conversely, the significantly positive *F*_*IS*_ for Large Mikana is indicative of a heterogyzote deficit and may be related to locus-specific underdominant selection (Miyamoto et al., unpubl. manuscript).

### Standard statistics

Although the six groups were initially treated as separate for the calculation of summary statistics, they were ultimately combined according to our series of four tests for population number and ongoing field observations with red colobus at KNP (see below). The combined dataset was checked for genotyping errors with MICRO-CHECKER, version 2.2.3 (van Oosterhout et al. 2004). Estimates of within-group diversity [observed and effective numbers of alleles (*n*_*a*_ and *n*_*e*_) and observed and expected heterozygosity (*H*_*o*_ and *H*_*e*_)] were calculated with POPGENE, version 1.3.1 (Yeh and Boyle 1997). Each locus was tested against its Hardy–Weinberg expectations with the G-test of POPGENE. Each pair of loci was assessed for genotypic disequilibrium with the G-test and 4,500 permutations in FSTAT, version 2.9.3 (Goudet 2002). Wright's *F*_*IS*_ was estimated for each locus with 10,000 randomizations using FSTAT and across all loci with 10,000 bootstrap replications in GDA, version 1.1 (Lewis and Zaykin 2002). To correct for multiple comparisons, a sequential Bonferroni correction was applied in all sample-wide series of tests (Rice 1989).

### Population number

Three tests were conducted to determine whether the six groups make up a single interbreeding population or multiple separate populations. The first approach relied on estimates of *R*_*ST*_ and *4Nm* as calculated with RSTCALC, version 2.2 (Goodman 1997) for all six groups and between all 15 pairs of groups. The significance of the *R*_*ST*_ estimates was determined with 10,000 permutations. The second approach used STRUCTURE, version 2.3.3 (Pritchard et al. 2000) and an admixture model with correlated allele frequencies among populations. The number of populations (*K*) was varied from one to six (one population per group). Each a priori setting of *K* was run 10 independent times with each run consisting of 1 × 10^8^ generations and a burn-in of 1 × 10^7^. Results were then summarized with STRUCTURE HARVESTER version 0.6.92 (Evanno et al. 2005).

Finally, we used log Bayes Factor (ln BF) testing with MIGRATE version 3.1.6 (Redelings and Suchard 2005; Beerli and Palczewski 2010). We used a sequential hierarchical approach for the testing of simpler to more complex models with ln BF. If the ln BF test for the current simpler model was decisive, we stopped there; otherwise, we would continue with a comparison of the more complex model to one with an extra population, Θ, or *4Nm*. For this analysis, we began with a comparison of the simplest one-population model (with its one free parameter, Θ) to its closest competitor with two free parameters (the two-population model with mean Θ and average *4Nm*). The two groups for the two-population model were chosen according to the longest internal branch of their neighbor-joining tree as generated with the Δμ^*2*^ pairwise distances of the six groups (Goldstein et al. 1995). The two-population model assumed a single mean Θ for both groups and symmetrical migration between them (i.e., an average *4Nm*). The calculation of log marginal likelihoods (ln mL) for the two models was done using thermodynamic integration and its Bezier approximation (Beerli and Palczewski 2010). These calculations relied on static heating with the temperatures of the three heated chains set at 1.5, 3.0, and 100,000. Each hot and cold chain was run for 1 × 10^8^ generations per locus with samples taken from the cold chain every 2,500 steps after a 10% burn-in. Uniform priors of 0–50 and 25–400 were used for Θ and *4Nm*, respectively. Three independent runs under both the one- and two-population models were performed to ensure the convergence of the final results. All MIGRATE analyses were done using the Brownian approximation of the SMM. Rate variation among the loci was accounted for by the incorporation of mutation rate modifiers that were calculated according to the number of observed alleles.

### Population size changes

We performed four tests to check for historical changes in population size. The first approach relied on BOTTLENECK, version 1.2.02 using the one-tailed Wilcoxon test for heterozygote excess and the module shift descriptor of the allele frequency distribution (Luikart et al. 1998). The one-tailed test was used for the former to increase the power of detecting a heterozygote excess, which is indicative of a recent population bottleneck. The underlying distribution of *H*_*o*_ in the Wilcoxon test was generated with 10,000 simulations under the two-phase mutation (TPM) model of microsatellite evolution. In this model, most mutations change the allele length by a single step, whereas a small proportion changes the allele size by two or more steps (Di Rienzo et al. 1998). The variance among multiple steps in the BOTTLENECK simulations was set to 12 as recommended by Piry et al. (1999). Then, M_P_VAL (Garza and Williamson 2001) was also used to test for a recent bottleneck. We tested the significance of the *M* ratio with 10,000 simulations under TPM in M_P_VAL. The proportion of single-step mutations and mean step size in the M_P_VAL simulations were set to 0.88 and 2.8, respectively, following the empirical recommendations of the program's authors. In the BOTTLENECK and M_P_VAL analyses, “recent” refers to a bottleneck where (e.g.) the population has decreased within the last 250 generations (i.e., 1,250 years for red colobus given a generation time of 5 years; see below) to *N*_*e*_ of 50 (Cornuet and Luikart 1996; Luikart et al. 1998). The recommended settings of Piry et al. (1999) and Garza and Williamson (2001) were adopted for the BOTTLENECK and M_P_VAL simulations, because they are empirically based on a number of different studies.

The second approach used MSVAR, version 1.3 to estimate the starting time (*t*) for a change in the ancestral (*N*_*1*_) to current (*N*_*0*_) population sizes (Beaumont 1999). The ratio of *N*_*0*_ to *N*_*1*_ (*N*_*0*_/*N*_*1*_ or *r*) was also calculated from their Markov chain Monte Carlo (MCMC) samples. The MSVAR analyses were performed with the exponential model of population growth and a generation time of 5 years for red colobus as estimated from their ages at sexual maturity (Struhsaker pers com.). Each analysis was run for 2 × 10^10^ generations with samples taken every 500,000 steps after a 10% burn-in. The log_10_ means and variances for the hyperprior means of *N*_*0*_, *N*_*1*_, and *t* were set to (3.6, 1.0), (3.6, 1.0), and (5.0, 1.0), respectively (other prior and hyperprior settings were also tried; see below). The log_10_ means and variances for their hyperprior variances were all set to (0.0, 0.5). Three independent runs were performed to ensure convergence.

The final approach estimated *N*_*e*_ through time using EBSP (Heled and Drummond 2008) in BEAST version 1.5.4. The Extended Bayesian Skyline Plot is a non-parametric piecewise model, which estimates the population function directly from the data instead of fitting a parametric model (e.g., exponential growth) specified a priori. Unlike the original Bayesian Skyline Plot (Drummond et al. 2005), EBSP combines information from multiple loci and estimates the number of population size change points, which is specified by the user as a fixed value in the Bayesian Skyline Plot. The analysis was performed with 12 microsatellite models (Sainudiin et al. 2004; Table 2), with four independent runs carried out for each implemented model (Wu and Drummond 2011). The observed maximum length of any allele was 33 repeats, so we assumed an upper bound of 35. The average mutation rate of all loci was fixed to one, but the relative rates were estimated to account for rate variation across the loci. At time point *t*_*j*_*,* θ_*j*_ was assumed to come from an exponential distribution with an unknown mean parameter (φ), to which a one-on-x prior was applied. A Poisson prior with mean λ = 0.6931 was used for the number of changes (ψ) in the population history (Heled and Drummond 2008). Each analysis was based on 8 × 10^8^ generations with samples taken every 4 × 10^5^ steps after an appropriate burn-in of 10–15%.

**Table 2:** Twelve models of microsatellite evolution

Mutation Model^a^	Ln marginal likelihood (Ln mL) b	Ln Bayes factor^c^ (Ln BF)	Mean coalescent time (*T*_COAL_)^d^	Model description^a^
PU2^e^	−942.14	0	98,000 (37,000–176,000)	Proportional-rate, unbiased, two-step
EC2	−946.28	4.15	105,000 (40,000–183,000)	Equal-rate, constant-bias, two-step
EL2	−950.43	8.29	121,000 (51,000–212,000)	Equal-rate, linear-bias, two-step
PC2	−951.90	9.77	114,000 (41,000–214,000)	Proportional-rate, constant-bias, two-step
PU1	−957.99	15.86	153,000 (61,000–267,000)	Proportional-rate, unbiased, one-step
EU2	−969.63	27.50	107,000 (42,000–182,000)	Equal-rate, unbiased, two-step
PC1	−980.49	38.36	163,000 (62,000–286,000)	Proportional-rate, constant-bias, one-step
EU1	−987.95	45.81	158,000 (69,000–277,000)	Equal-rate, unbiased, one-step
EC1	−992.24	50.11	154,000 (70,000–257,000)	Equal-rate, constant-bias, one-step
PL2	−997.90	55.77	129,000 (51,000–235,000)	Proportional-rate, linear-bias, two-step
PL1	−1006.89	64.76	197,000 (71,000–349,000)	Proportional-rate, linear-bias, one-step
EL1	−1023.05	80.92	180,000 (76,000–310,000)	Equal-rate, linear-bias, one-step

a All models have been described in Sainudiin et al. (2004), with the exception that we use a simple logistic regression (rather than simple linear regression) to model a directional bias of mutation (C and L below). These 12 models vary (1) according to whether the mutation rate is independent of allele length (E = equal, rate is independent of length and P = proportional, rate is proportional to microsatellite length); (2) according to whether the probability of a contraction is equal to that of an expansion (U = unbiased, equal probabilities for the two, C = constant bias, the probabilities of contraction and expansion are not equal, but remain constant and are independent of allele length, and L = linear bias, unequal probabilities for the two events, which now depend on allele length), and (3) according to whether a mutation can change the length of an allele by more than one repeat (1 = single step, only mutations of a single repeat are allowed and 2 = multi-step, a mutation can change the length of an allele by one or more repeats). Thus, the PU2 model accounts for a microsatellite mutation rate that varies according to allele length, equal rates of contraction and expansion, and mutations of multiple, as well as single, repeats.

b The 12 models are listed from best to worst according to their ln mL.

c Relative to the best (PU2) model.

d 95% HPD in parentheses.

e The PU2 model receives >98% of the relative probability (i.e., the total marginal likelihood for all 12 models).

We next completed BEAST analyses of the microsatellite data, assuming a model of constant population size. These constant population size analyses using BEAST were compared to the EBSP runs to test further the hypothesis of a stable population of red colobus at KNP. These additional BEAST analyses were conducted with the best-fit microsatellite model from the EBSP comparisons (PU2; see Table 2) as well as with the EU1 model, which best approximates the SMM. We applied coalescent priors to all sampled genealogies of the microsatellites, which assume a constant population size that is shared across all loci. The common population size parameter was given the one-on-x prior, f_X(x) = 1/x, which is the Jeffrey's prior for a constant population size. Log BF were then used to compare the fits of the constant population size model to those of EBSP as described in the next paragraph.

Log Bayes Factors between each pair of microsatellite mutation and/or population (constant population size vs. EBSP) models were computed by TRACER version 1.4.1 (Rambaut and Drummond 2007) for model selection. Here, the harmonic means of the sampled log likelihoods (ln L) were used to calculate the ln mL of the models. The models were then ranked from best to worse according to their ln BF.

The MCMC sampling in each run with STRUCTURE, MIGRATE, MSVAR, and BEAST was monitored in TRACER to confirm its mixing and convergence. Different run conditions, priors, and/or hyperpriors were also examined in addition to those reported above for the final production runs. These experiments were performed to establish the final settings of the production runs (e.g., lengths, burn-in, and underlying distributions) and to verify that small changes did not result in significantly different estimates. The log files for the final production runs and for these additional experiments are available upon request from the corresponding author. Readers can inspect these log files with TRACER to further assess the posterior probabilities, mixing, and convergence of the different population, substitution, and MCMC parameters.

For these analyses, we used the mutation rate, 5 × 10^−4^, calculated from human data using detailed microsatellite mutation models (Whittaker et al. 2003). However, another mutation rate, 1 × 10^−4^, has also been used in microsatellite studies of the population genetics of humans and other primates (Ruiz-Garcia 2005; Payseur et al. 2011). Changing our mutation rate would not change the overall findings of the study, but would increase the coalescent times (*T*_*COAL*_ defined by the height of the tallest tree at each step of the MCMC) and *N*_*e*_ by a factor of five. We chose to present our results using the Whittaker et al. (2003) rate because it supports smaller, and thus, more conservative estimates of central importance to our conclusions and to conservation biology. For example, these results offer smaller estimates for the age of the red colobus population, and thereby Kibale forest, and for minimum viable population size.

## Results and discussion

### Basic statistics

The original microsatellite dataset consisted of 11 loci from 85 red colobus living in KNP. Locus *D17S1290* was the only locus with a significant *F*_*IS*_ after Bonferroni correction and it was significantly positive (indicative of a heterozygote deficit). This locus was also the only locus with both a significant number of null alleles and a homozygote excess due to scoring errors according to MICRO-CHECKER. Due to these discrepancies, this locus was excluded from all further analyses, including the following summary statistics (Table 1). An average of 7.70 alleles was scored from each locus, and heterozygosities averaged 0.70 for *H*_*0*_ and 0.72 for *H*_*e*_. None of the 45 pairs of loci were in genotypic linkage disequilibrium after Bonferroni correction. In turn, only one locus (*D20S206*) deviated significantly from Hardy–Weinberg expectations, but this departure was not due to either a heterozygote deficit or excess according to its non-significant *F*_*IS*_. This result of a significant Hardy–Weinberg test, but insignificant *F*_*IS*_ was due to the existence of a heterozygote excess for the genotypes of some alleles, but a heterozygote deficit for others.

### Number of populations

All three approaches for examining the number of populations of red colobus from KNP supported a single population. First, the *R*_*ST*_ and *4Nm* estimates across all six groups were negative according to RSTCALC (−0.01 and −25.28, respectively), with the former not deviating significantly from zero according to its permutation test (*P* > 0.10). None of the *R*_*ST*_ for the 15 pairwise comparisons between groups were significant (after Bonferroni correction). Thus, the RSTCALC results reveal no significant genetic differences among the six groups, and indicate that the six groups can be considered as members of one panmictic population.

**Figure 3:**
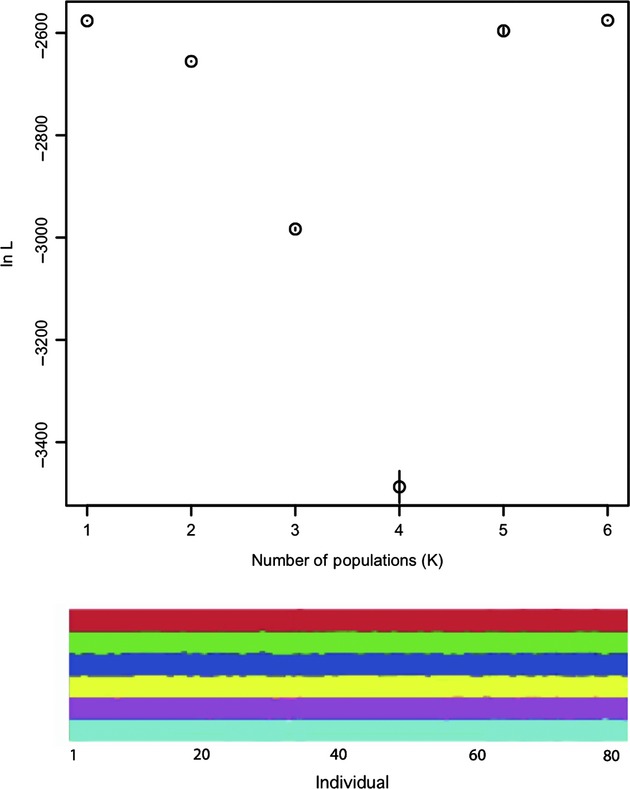
(Above) Mean ln L (circles) and their standard deviations (vertical lines) for *K* = 1 to 6 populations as obtained with 10 independent runs per *K* with STRUCTURE. (Below) Relative probabilities of assigning the 85 individuals of red colobus to six (differently colored) populations. These STRUCTURE results clearly show that little to no support exists for the assignment of the 85 individuals to separate distinct populations.

Second, mean ln L followed a U-shaped pattern as *K* (number of populations) was increased from 1 to 6 in the STRUCTURE analysis (Fig. 3). This pattern of elevated mean ln L for larger values of *K* has been reported by the authors of STRUCTURE (Pritchard and Wen 2003) as well as by others (Evanno et al. 2005). It has been attributed to inflated support for values of *K* greater than the true number of populations. Thus, of greater importance is the failure of the STRUCTURE analysis with *K* = 6 to clearly assign the 85 individuals of red colobus to separate populations (Fig. 3). This failure, coupled with the earlier warnings of others, indicates that one population is more likely than two or more for the red colobus of KNP.

Finally, the longest internal branch in the unrooted neighbor-joining tree with Δμ^2^ distances divided the six groups into two larger groups of Dura, K30, and Large Mikana versus Mainaro, Sebatoli, and Small Camp (see Fig. 2 for group names). The ln mL for the one-population model was −8,196.98 according to MIGRATE. Conversely, the ln mL for the two-population model (with its two groups as defined above by the neighbor-joining tree) was –26,261.91. Thus, the two models differed by a ln BF of 18,064.93. In the statistics literature, a ln BF of >5 is generally considered to be “decisive” for one model over another (Kass and Raftery 1995). Following this literature, the one-population model is thereby taken as strongly favored over the two-population model with mean Θ and symmetrical migration. Given this selection of the simpler one-population model, we stopped there. Otherwise, we would have continued with an evaluation of a two-population/three parameter model (e.g., one with asymmetrical migration and mean Θ). Taken together, the RSTCALC, STRUCTURE, and MIGRATE results suggest high levels of gene flow throughout the park indicating that the red colobus of KNP is one large interbreeding population. These results fit with observational data from the field. For example, we have observed three female dispersals and one male dispersal in a year between the Large Mikana and Small Camp groups. On the basis of the genetic and field evidence, all groups were analyzed as a single population in the calculation of the summary statistics (Table 1) and in the following analyses of population fluctuation.

### Population fluctuations

The results from BOTTLENECK and M_P_VAL showed little evidence of a recent bottleneck for the red colobus in KNP. The one-tailed Wilcoxon test for heterozygote excess was not significant (*P* = 0.410) and the allele frequency distribution was L-shaped according to BOTTLENECK. The *M* ratio was calculated as 0.839, which was not significant (*P* = 0.303) according to its simulations in M_P_VAL. This large *M* ratio agrees with the empirical estimates of other species with stable population sizes (Garza and Williamson 2001).

**Figure 4:**
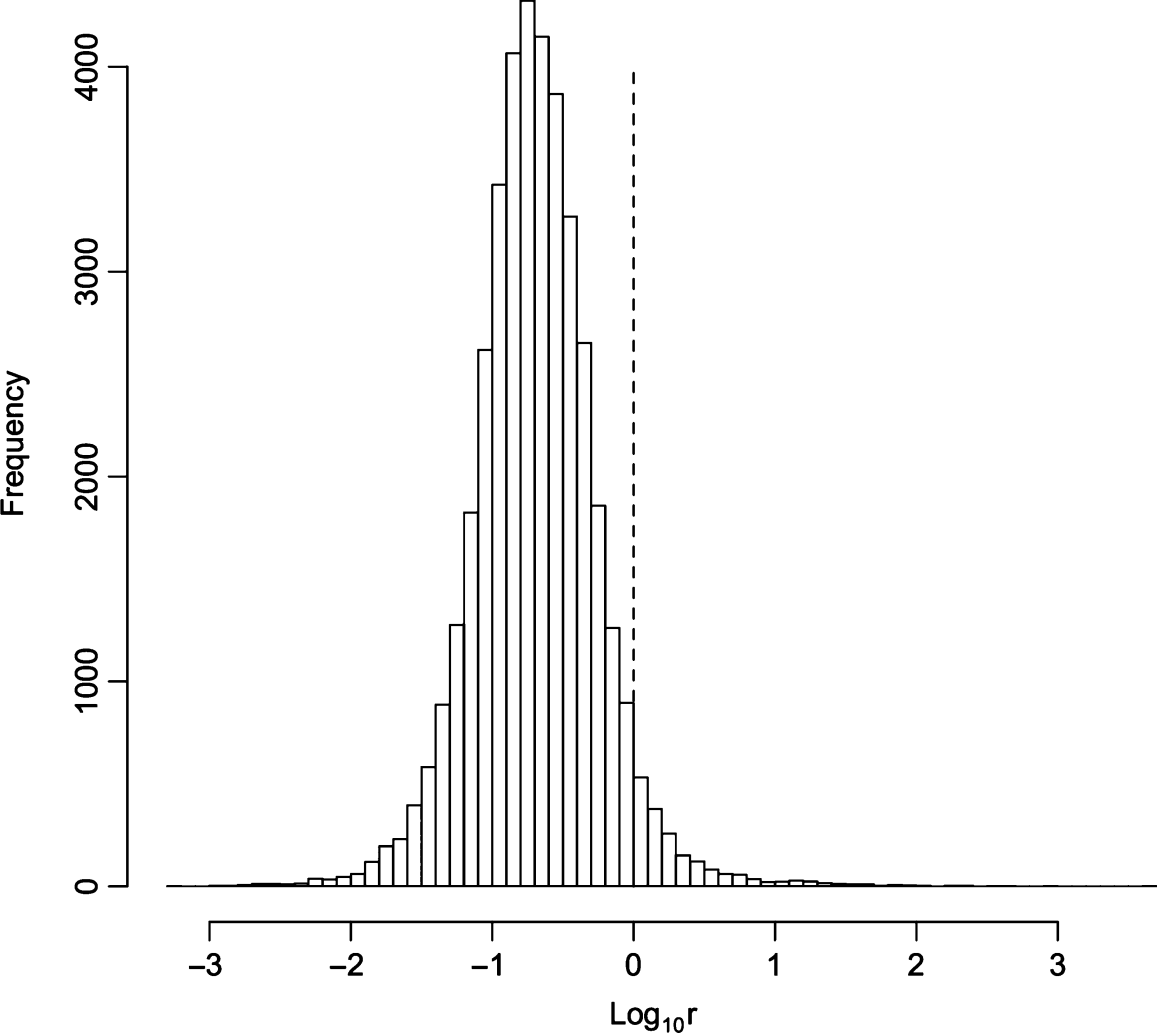
Distribution of log_10_ *r* (log_10_ *N*_*0*_/*N*_*1*_) summarizing the 36,000 MCMC samples from MSVAR. *N*_*1*_ represents the past population size and *N*_*0*_ represents the size of the current population. The upper boundary of our 95% credibility interval overlaps log_10_ *r* = 0 as shown by the vertical gray line. This is where *N*_*0*_ = *N*_*1*_*,* which is indicative of no population size change over time. The log10 *r* estimates of this histogram support a model of population decline over growth by a ln BF of 20.518 (Storz & Beaumont 2002). Conversely, our study concludes in favor of a constant population given that log10 *r* = 0 falls within the 95% credibility interval of these estimates.

The MSVAR results were less clear. The averages and 95% highest posterior densities (HPD) for log_10_ *N*_*0*_ (current *N*_*e*_), *N*_*1*_ (ancestral *N*_*e*_), *r* (*N*_*0*_/*N*_*1*_), and *t* (starting time of the population size change) were (3.22, 2.60–3.75), (3.92, 3.24–4.67), (−0.70, −1.58–0.16), and (4.18, 2.54–5.97) according to the 36,000 MCMC samples of MSVAR. Thus, the 95% HPD for log_10_ *r* included zero, which is the critical point for *N*_*0*_ = *N*_*1*_. This critical point fell within the right tail of the posterior distribution at *P*(two-tailed) = 0.092 (Fig. 4). Conversely, the transformed linear means and 95% HPD for *r* and *t* were (0.20, 0.03–1.45) and (15,136, 347–993,254 years ago), which suggests a decline in *N*_*e*_ of ∼80% over the last ∼15,000 years. We also note that Storz and Beaumont (2002) described a ln BF test, which compares how well a model for population contraction fits the data relative to one for expansion. Nevertheless, because our 95% HPD for the log_10_ *r* included zero, we cannot reject the hypothesis of a constant population. Furthermore, the estimated mean and median for the start of this decline (∼15,000 years ago) falls within a period when tropical forest regions were expanding as earth entered its current interglacial (Castañeda et al. 2009). Thus, when coupled with the non-significant deviation of log_10_ *r* from zero, we cannot exclude a constant population hypothesis.

**Figure 5:**
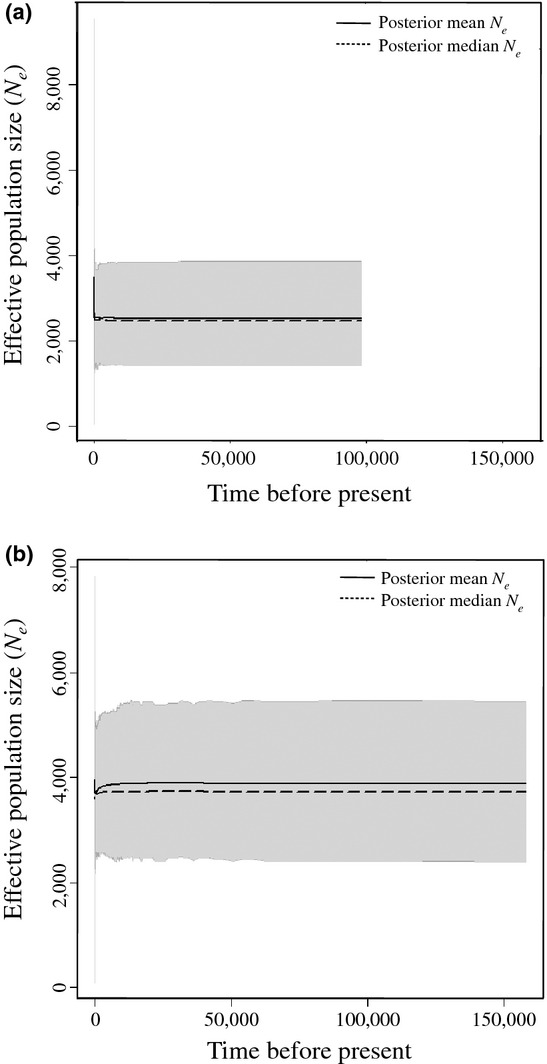
Extended Bayesian Skyline Plots for the 10 microsatellite loci using the PU2 (proportional-length, unbiased, two-step) model (*a*) and EU1 (equal-rate, unbiased, one-step) model (*b*). These skyline plots are extended to their mean coalescent times (*T*_*COAL*_) on the right end of the graph. The gray shading corresponds to the 95% HPD around the mean *N*_*e*_. PU2 exhibits the best fit to the data, whereas EU1 approximates the SMM (Table 2). Both models (as do the other ten) show similar skyline plots with flat lines that correspond to a stable population size over time coalescing between ∼100,000 and 200,000 years ago.

The EBSP for all models show a flat line for *N*_*e*_ since mean *T*_*COAL*_ (Fig. 5). The posterior probabilities of the demographic.popSizeChange was the highest when it was 0 and all 95% credible intervals included 0 for all models suggesting no population size change in the past. Of these 12 models for microsatellite evolution, the proportional-length, two-step (PU2) model had the best fit to our dataset (Table 2). This model was supported over its closest competitor, the equal-length, constant-size, two-step (EC2) model, by a non-decisive ln BF of 4.15 (Kass and Raftery 1995). The poorest model was the equal-rate, linear-size, single-step model (EL1; ln BF = 80.92 vs. PU2; Table 2). The mean *T*_*COAL*_ was estimated between 98,000 and 197,000 years ago among the 12 models, with the best model (PU2) predicting 98,000 years ago. Similarly, MIGRATE, using the Brownian approximation of the SMM and the one-population model, calculated a mean *T*_*COAL*_ of 112,848 years ago (standard deviation = 71,414). The ln mL for the constant population size analyses with the PU2 and EU1 models were −943.44 and −988.68, respectively, and differed from those of their EBSP counterparts with PU2 and EU1 by ln BF of 3.68 and 2.08, respectively (Table 2). These small ln BF are non-decisive according to Kass and Raftery (1995). Thus, the near-equal fits of both constant population size models confirm that the EBSP graphs are indicative of a stable population for the red colobus in KNP.

The EBSP approach is powerful and flexible, because it is not constrained a priori to a specific model of historical population size change (Heled and Drummond 2008). Thus, the EBSP recovery of a constant population for the red colobus in KNP is derived from the data rather than from the a priori selection of a particular growth model. This support for a constant population is based on the maximum clade credibility (MCC) genealogies of each microsatellite locus (Appendix A2), which show a standard coalescent pattern of increasing waiting times between coalescences as one works backward in time (Wakeley 2008). Thus, relatively few internodes of each MCC gene genealogy occur more than >10,000 years ago. It is important to recognize that this paucity of old internodes does not reflect a lack of support for a constant population with mean and median *T*_*COAL*_ of ∼100,000 years ago. Instead, this paucity of old internodes contributes to the signal for a constant population, rather than for some other model such as for an expanding population, where the coalescences are expected to cluster closer to the base of each gene genealogy (Page and Holmes 1998; Hein et al. 2005). However, in keeping with our conservative approach, we limit our estimate of the population stability in red colobus to at least ∼40,000 years ago (i.e., to the lower boundary of the 95% HPD of *T*_*COAL*_ for the PU2 microsatellite model; Table 2).This conservative treatment of the duration of the population stability is also warranted given that *T*_*COAL*_ is an estimate of the coalescent time of gene genealogies and not necessarily the most recent common ancestor of the population (Hein et al. 2005).

**Figure 6:**
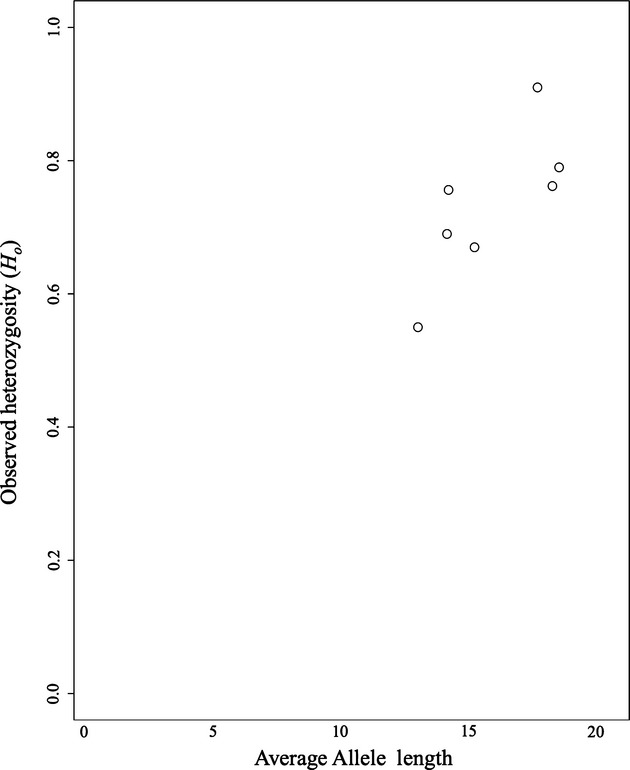
Significant positive correlation between *H*_*o*_ and average allele length for seven tetranucleotide loci (Spearman Rank Correlation, *P* = 0.033). The average allele lengths for each locus are weighted by their allele frequencies. This comparison does not include the three dinucleotide loci of the full dataset.

The evolution of microsatellites is complex because it is characterized by various mutational biases (Goldstein and Pollock 1997). A common property is the positive relationship between allele length and mutation rate, which is evident in our data (Fig. 6). Such complexities call for the development and use of more realistic evolutionary models for microsatellites, such as the PU2 model (Table 2). Nevertheless, the fact that even simple models (e.g., EU1 which approximates the SMM) produce skyline plots similar to those of complex models (Fig. 5) suggests that simpler models can also be of value, particularly when dealing with large microsatellite datasets that are computationally intensive. The maximum clade credibility trees scaled to years for the 10 loci for models PU2 and EU1 are in Appendix A2.

### Effective and census population sizes

Mean *N*_*e*_ varied among the EBSP graphs for the 12 microsatellite models from ∼2,500 for the two best models (PU2 and EC2) to ∼4,000 (Fig. 5). The BEAST analyses with a constant population size model supported mean *N*_*e*_ of 2,517 (95% HPD of 1,347–3,710) and 3,677 (2,561–4,857) for the PU2 and EU1 models, respectively. In turn, the one-population analysis with MIGRATE (which also assumes a constant population size) supported mean *N*_*e*_ of 4,055 (2,978–5,426). Collectively, these different analyses of population size agree on a mean *N*_*e*_ of ∼2,500 to 4,000 for the red colobus in Kibale.

Using this range of 2,500–4,000, we estimate the *N*_*e*_/*N*_*c*_ ratio to be ∼0.15 to 0.25 for this population. These numbers are lower than the previous *N*_*e*_/*N*_*c*_ estimate of ∼0.35 for the red colobus population in KNP (Struhsaker and Pope 1991). However, this previous *N*_*e*_/*N*_*c*_ estimate is based on older census techniques and calculations of male and female reproductive success, and is currently thought to be an overestimate (T. Struhsaker pers com.). Importantly, a number of factors are thought to reduce *N*_*e*_ with respect to census estimates: skewed sex ratio, overlapping generations, and variation in adult reproductive success (Frankham 1995). The red colobus at KNP are characterized by all of these demographic factors. They exhibit a skewed sex ratio of one breeding male to every 1.3 breeding females (Struhsaker and Pope 1991). Red colobus also have overlapping generations, their overall lifespan is thought to be around 9.3 years (Pope 1996) with an overall reproductive lifespan of 8.8 years, and adults average one birth every 2 years (Struhsaker and Pope 1991). Combined, these factors likely explain the low *N*_*e*_/*N*_*c*_ ratio found in this population of red colobus.

In conservation biology, a critical *N*_*e*_ of ∼500 to 1,000 is often cited as necessary for the genetic security and long-term survival of a population (Franklin and Frankham 1998). However, other studies have argued from the perspective of the nearly neutral theory that a minimal viable population size of ∼1,000 to 5,000 is more appropriate (Lynch and Lande 1998). Our *N*_*e*_ estimates for the red colobus in Kibale (∼2,500 to 4,000) overlap with the latter values, and thereby suggest that these larger *N*_*e*_ are important for the genetic integrity and long-term viability of populations, similar to other recent studies (Flather et al. 2011).

### History of red colobus and Kibale forest

Red colobus (*Procolobus*[*Piliocolobus*]) are found throughout most of equatorial Africa in small, mostly declining populations (Fig. 2). The morphotype, *P. [Piliocolobus] rufomitratus* belongs to a central/eastern African clade of red colobus (Struhsaker 1981b; Grubb et al. 2003) that radiated around 1.4 million years ago (Ting 2008). *Procolobus [Piliocolobus] rufomitratus* likely split from other *Piliocolobus* sometime after 600,000 years ago (Ting 2008), and our results suggest that the KNP population of red colobus has remained stable for ∼40,000 years or more.

Red colobus are an important indicator species of their habitat, such that evidence of a population size change can be interpreted as a change in their habitat (Struhsaker 2005). Our results suggest that these monkeys have been in a forest approximately the same size as KNP during at least the last ∼40,000 years as changes in the available forest environment, or population movement, would have led to a corresponding change in population size. Furthermore, in recent years, no population size change in red colobus has been detected from 1970 to 2006 suggesting the forest is at carrying capacity for the red colobus (Chapman et al. 2010a,b). Thus, the most parsimonious hypothesis is that the forest of Kibale National Park has been persistently occupied by this population of red colobus for the duration of their population stability. This suggests that the forest has served as a refuge for tropical rainforest species during the last ∼40,000 years and possibly even longer.

The alternative hypothesis, that this population of *P. rufomitratus* occupied a forest other than KNP during this time and then moved *en masse* to this area, is less likely for several reasons. The lack of a dramatic drop in population size does not support a recent founder event or the possibility that these red colobus are the remnants of a larger and more widely dispersed population. If these events occurred, they must have done so earlier than ∼40,000 years ago. It is also unlikely that this population of red colobus migrated *en masse* from another forest similar in size and environment sometime during the Late Pleistocene without any significant change in effective population size (e.g., bottleneck or founder event). Therefore, we conclude that the population stability of red colobus in KNP during the Late Pleistocene reflects similar long-term stability of the Kibale forest environment.

East Africa has experienced many paleoenvironmental and anthropogenic changes over the last 100,000 years. This geographic region has undergone many long periods of extreme drought during the Late Pleistocene, first from ∼135,000 to 70,000 years ago (Scholz et al. 2007), and again during the last glacial maximum ∼18,000 years ago when Lake Victoria completely dried up (Stager and Johnson 2008) and regional vegetation changed to scrub and montane grasslands (Jolly et al. 1997). During these periods, a major Central Refuge for tropical forest species existed to the west of Kibale forest over the Rwenzori Mountains in the neighboring Democratic Republic of the Congo (Hamilton 1976; Jolly et al. 1997; Wronski and Hausdorf 2008). Similar periods of environmental variability existed during the Holocene and are evidenced by a population crash of Ugandan and Kenyan buffalo indicative of a drought ∼4,500 years ago (Heller et al. 2008). There is further evidence of anthropogenic impacts to the forests surrounding Kibale forest starting with the arrival of the Bantu-speaking people ∼2,300 years ago and through subsequent shifts in their settlement patterns (Taylor et al. 1999).

Despite this variability, there is evidence that upper montane forests may have persisted during these climatic cycles (Jolly et al. 1997). Critically, Kibale forest is considered a separate habitat patch that persists due to its favorable mid-elevation topography and humidity (Struhsaker 1981a; Jolly et al. 1997; Moore 1998; Wronski and Hausdorf 2008). Our results for a constant red colobus population in KNP corroborate the hypothesis of a stable Kibale forest, despite the known environmental and human changes in East Africa during the Late Pleistocene and Holocene.

Our results further suggest that a tropical rainforest patch of only ∼795 km^2^ (i.e., the size of KNP) is sufficient to support a long-term viable population of red colobus. This conclusion is important given the rapid rate at which their habitat is disappearing and the lack of knowledge of what makes a suitably sized conservation area. However, this estimate of a minimal habitat size refers specifically to red colobus because other species differ in their ecology and behavior and will have other habitat requirements (e.g., it is known that a territorial top predator such as a leopard needs a much larger habitat for the long-term survival of its population).

We now call for critical tests of our hypotheses for an old and stable KNP population of red colobus and, thus, Kibale forest. Palynological studies from throughout KNP are needed to critically test the hypothesis of a continuous old tropical rainforest of approximately the same size as that currently found in the park (Jolly et al. 1997). There has been no known connectivity between the red colobus of KNP and any other group since the 1950s. However unlikely, population genetic studies are now needed to test whether gene flow is occurring or has occurred with these other populations in East Africa. These studies are particularly important given that tropical rainforest in East Africa was much more widespread in the past (e.g., during the Holocene Climatic Optimum ∼6,000 to 10,000 years ago; Jolly et al. 1997). They are also significant in light of recent simulation studies (e.g., Chikhi et al. 2010; Peter et al. 2010), which show that immigration can result in a false signal of a population decline (i.e., the ∼80% decrease at ∼15,000 years ago as suggested by MSVAR). Additional simulations are also now needed to validate the efficiency and robustness of EBSP to recover older population size changes with microsatellite data (Wu and Drummond 2011). Such critical tests and experiments are necessary for the final acceptance of our conclusions for an old and stable population of red colobus and Kibale forest.

Biodiversity is lost at an astonishing rate each year, particularly in the tropics where deforestation is rapidly extinguishing species habitats. In Uganda, it is estimated that 85% of the forests have disappeared (Howard et al. 2000). Wise conservation efforts over the last 40 years in Kibale National Park have ensured that this area has persisted as a protected National Park (Box et al. 2008). Our research suggests that this forest has been stable for at least the last ∼40,000 years, during which it likely served as an important sanctuary for tropical rainforest species in East Africa. Furthermore, our findings indicate that species with effective population sizes of ∼2,500 to ∼4,000 may be able to persist long term in stable environments, thereby providing hope for other species in decline or with patchy distributions.

Kibale forest offers a rich source of information about Pleistocene refugia, which increases its importance as a major center for conservation and scientific research. With continued wise management, KNP can likely continue to serve as a critical refuge for these species well into the future.

## Data accessibility

The raw data used in the analyses are available online in Appendix A1. The maximum clade credibility trees for the sampled genealogies of each locus (as obtained from the EBSP analysis using the PU2 and EU1 models; Fig. 5) are available in Appendix A2.

## Supporting Information

**Appendix A1.** Genotypes for 85 red colobus from six groups in Kibale National Park (KNP). Missing data are indicated by an X. Group locations in KNP are given in Figure 1; SM – Small Camp, LM – Large Mikana, K30 – K-30, DUR – Dura, SEB – Sebatoli, and MAIN – Mainaro.

**Appendix A2.** The maximum clade credibility trees for each of the 10 loci for two models of molecular evolution, (I) our best-fit model – PU2 and (II) EU1 the model that most approximates a stepwise mutation model. The time scale is in terms of years.
