# Supplementary material for: Primate DNA suggests long-term stability of an African rainforest
Source: Ecol Evol. 2012 Oct 9;2(11):2829–42. doi: 10.1002/ece3.395 (PMC3501634; doi:10.1002/ece3.395)
Supplement: Supplementary file 1 [file ece30002-2829-SD1.doc]

**APPENDIX A**

Genotypes for 85 red colobus from six groups in Kibale National Park (KNP). Missing data are indicated by an X. Group locations in KNP are given in Figure 1; SM – Small Camp, LM – Large Mikana, K30 – K-30, DUR – Dura, SEB – Sebatoli, and MAIN – Mainaro.

| **ID** | **Group** | **D14S306** | | **D3S1766** | | **D2S1399** | | **D7S1817** | | **D20S206** | | **D8S260** | | **D8S165** | | **D1S207** | | **D17S1290** | | **C2A** | | **D5S1457** | |
| --- | --- | --- | --- | --- | --- | --- | --- | --- | --- | --- | --- | --- | --- | --- | --- | --- | --- | --- | --- | --- | --- | --- | --- |
| 27 | SM | 176 | 188 | 232 | 245 | 155 | 175 | 149 | 169 | 150 | 154 | 194 | 200 | 157 | 159 | 153 | 155 | 174 | 174 | 284 | 296 | 128 | 132 |
| 999 | SM | 180 | 184 | 240 | 240 | 167 | 171 | 149 | 165 | 146 | 150 | 194 | 194 | 157 | 159 | 143 | 171 | 178 | 178 | 284 | 288 | 136 | 144 |
| RGS | SM | 172 | 176 | 232 | 249 | 151 | 171 | 161 | 161 | 154 | 154 | 194 | 196 | 157 | 157 | 165 | 165 | 174 | 174 | 284 | 284 | 132 | 144 |
| BBS | SM | 180 | 184 | 232 | 245 | 167 | 171 | 149 | 157 | 150 | 154 | 194 | 194 | 157 | 159 | 155 | 165 | 174 | 174 | 284 | 296 | 136 | 144 |
| BGS | SM | 176 | 176 | 232 | 240 | 151 | 167 | 149 | 157 | 142 | 154 | 194 | 194 | 157 | 159 | 165 | 169 | 174 | 174 | 284 | 284 | 136 | 140 |
| BRH | SM | 176 | 176 | 232 | 240 | 159 | 167 | 149 | 157 | 150 | 154 | 194 | 194 | 157 | 159 | 145 | 171 | 174 | 174 | 284 | 284 | 132 | 140 |
| BBH | SM | 180 | 188 | 227 | 240 | 167 | 171 | 161 | 169 | 138 | 138 | 194 | 194 | 157 | 157 | 165 | 167 | 174 | 174 | 284 | 284 | 132 | 144 |
| BSH | SM | 176 | 176 | 232 | 240 | 167 | 182 | 149 | 161 | 138 | 154 | 194 | 196 | 157 | 159 | 143 | 169 | 174 | 174 | 284 | 284 | 136 | 140 |
| SBH | SM | 176 | 184 | 240 | 240 | 159 | 167 | 161 | 165 | 138 | 154 | 194 | 194 | 157 | 157 | 143 | 143 | 174 | 174 | 284 | 284 | 140 | 144 |
| CSH | SM | 184 | 188 | 240 | 240 | 159 | 182 | 161 | 173 | 138 | 154 | 194 | 200 | 157 | 159 | 163 | 165 | 170 | 182 | 284 | 284 | 136 | 144 |
| CGS | SM | 172 | 176 | 227 | 232 | 155 | 171 | 161 | 165 | 150 | 154 | 194 | 200 | 157 | 157 | 143 | 143 | 174 | 174 | 284 | 288 | 140 | 144 |
| CRH | SM | 184 | 184 | 240 | 249 | 159 | 175 | 161 | 161 | 150 | 154 | 194 | 194 | 157 | 159 | 143 | 165 | 174 | 174 | 284 | 284 | 140 | 140 |
| RRS | SM | 184 | 184 | 245 | 245 | 171 | 182 | 149 | 161 | 138 | 150 | 194 | 194 | 157 | 159 | 153 | 165 | 174 | 174 | 284 | 288 | 140 | 144 |
| BEI | SM | 176 | 184 | 240 | 249 | 167 | 182 | 161 | 165 | 142 | 142 | 194 | 200 | 157 | 157 | 165 | 165 | 174 | 178 | 284 | 288 | 140 | 140 |
| CAT | SM | 172 | 184 | 232 | 249 | 167 | 182 | 161 | 165 | 142 | 154 | 190 | 194 | 157 | 157 | 143 | 165 | X | X | 288 | 300 | 140 | 144 |
| CSH | SM | 176 | 188 | X | X | 159 | 167 | 165 | 165 | 138 | 154 | 194 | 194 | 159 | 161 | 155 | 165 | X | X | 284 | 288 | 136 | 144 |
| DUP | SM | 172 | 176 | 227 | 249 | 171 | 182 | 161 | 165 | 142 | 154 | X | X | 157 | 157 | 143 | 165 | 174 | 174 | 284 | 284 | 132 | 136 |
| FATI | SM | 184 | 184 | 240 | 249 | 167 | 171 | 161 | 161 | 138 | 150 | 194 | 200 | 159 | 159 | 145 | 145 | 174 | 178 | 284 | 288 | 140 | 140 |
| FBTM | SM | 176 | 182 | 232 | 245 | 159 | 167 | 161 | 161 | X | X | 194 | 196 | 157 | 159 | 143 | 155 | 174 | 182 | 284 | 288 | 136 | 140 |
| FBTR | SM | X | X | 245 | 245 | 155 | 167 | 147 | 165 | 138 | 150 | 194 | 194 | 157 | 159 | 165 | 169 | 170 | 178 | 288 | 288 | 140 | 144 |
| FLIP | SM | 180 | 182 | 232 | 240 | 167 | 167 | 143 | 165 | X | X | 196 | 200 | 157 | 159 | 159 | 169 | 174 | 174 | 288 | 288 | 136 | 136 |
| FRED | SM | 172 | 184 | 227 | 253 | 162 | 175 | 161 | 165 | 138 | 150 | 194 | 194 | 159 | 159 | 165 | 165 | 174 | 174 | 288 | 296 | 132 | 144 |
| KEN | SM | 176 | 184 | 245 | 245 | 175 | 182 | 157 | 165 | 138 | 146 | 194 | 200 | 157 | 157 | 153 | 153 | 174 | 174 | 284 | 288 | X | X |
| **ID** | **Group** | **D14S306** | | **D3S1766** | | **D2S1399** | | **D7S1817** | | **D20S206** | | **D8S260** | | **D8S165** | | **D1S207** | | **D17S1290** | | **C2A** | | **D5S1457** | |
| LUP | SM | 184 | 188 | 240 | 240 | 159 | 182 | 143 | 165 | X | X | 194 | 194 | 159 | 159 | 143 | 143 | 174 | 174 | 284 | 284 | 132 | 136 |
| MCR | SM | 176 | 184 | 232 | 249 | 171 | 182 | 161 | 161 | 138 | 142 | 194 | 200 | 157 | 157 | 143 | 153 | 174 | 174 | 284 | 284 | 132 | 136 |
| MIS | SM | 176 | 180 | 232 | 240 | 159 | 167 | 165 | 177 | 0 | 0 | 196 | 200 | 157 | 159 | 151 | 163 | 174 | 174 | 284 | 288 | 140 | 144 |
| MKU | SM | 172 | 176 | 227 | 249 | 171 | 182 | 149 | 157 | 146 | 150 | 194 | 194 | 157 | 159 | 143 | 171 | X | X | 284 | 284 | 132 | 136 |
| MOM | SM | 180 | 188 | 240 | 245 | 159 | 182 | 161 | 165 | 142 | 150 | 194 | 196 | 157 | 159 | 165 | 171 | 170 | 182 | 284 | 307 | 140 | 144 |
| MSF | SM | 172 | 180 | 232 | 240 | 167 | 167 | 165 | 165 | 138 | 142 | 194 | 200 | 157 | 159 | 153 | 163 | 174 | 174 | 288 | 307 | 136 | 144 |
| MTD | SM | 176 | 184 | 240 | 249 | 171 | 175 | 165 | 173 | 142 | 142 | 194 | 200 | 157 | 157 | 153 | 165 | 174 | 174 | 284 | 284 | 140 | 140 |
| NATALI | SM | 172 | 184 | 227 | 253 | 159 | 167 | 157 | 143 | 138 | 150 | 194 | 194 | 157 | 159 | 167 | 167 | 174 | 174 | 284 | 288 | 132 | 144 |
| PAL | SM | 172 | 176 | 232 | 240 | 151 | 159 | 161 | 161 | 154 | 154 | 194 | 196 | 157 | 159 | 143 | 165 | 174 | 174 | 284 | 296 | 136 | 148 |
| STM | SM | 182 | 184 | 240 | 240 | 167 | 171 | 147 | 147 | 142 | 154 | 194 | 196 | 157 | 159 | 143 | 171 | 174 | 174 | 284 | 288 | 136 | 140 |
| SAF-TH | SM | 176 | 184 | X | X | 167 | 167 | 149 | 149 | 146 | 146 | 194 | 194 | 159 | 159 | 167 | 169 | 174 | 174 | 284 | 284 | 132 | 140 |
| TCF | SM | 176 | 180 | 240 | 249 | 171 | 182 | X | X | 129 | 129 | 194 | 194 | 157 | 157 | 143 | 155 | 174 | 174 | 284 | 288 | 140 | 140 |
| FST | SM | 172 | 184 | 227 | 240 | 155 | 171 | X | X | X | X | 194 | 200 | 157 | 157 | 155 | 165 | 174 | 174 | 284 | 284 | 140 | 140 |
| 996 | LM | 172 | 176 | 249 | 253 | 155 | 171 | 157 | 157 | 150 | 150 | 194 | 200 | 157 | 159 | 143 | 171 | 174 | 174 | 288 | 296 | 140 | 140 |
| 997 | LM | 172 | 184 | 232 | 249 | 167 | 175 | 161 | 165 | 150 | 154 | 194 | 200 | 157 | 159 | 143 | 169 | 174 | 174 | 284 | 300 | 136 | 144 |
| RSS | LM | 176 | 184 | 245 | 245 | 159 | 179 | 157 | 161 | 142 | 154 | 194 | 194 | 159 | 159 | 153 | 165 | 174 | 174 | 284 | 288 | 140 | 144 |
| RRH | LM | 176 | 176 | 245 | 245 | 167 | 171 | 149 | 173 | 142 | 154 | 194 | 196 | 157 | 157 | 165 | 171 | 174 | 174 | 284 | 296 | 136 | 144 |
| RBH | LM | 172 | 184 | 236 | 240 | 171 | 182 | 161 | 161 | 138 | 154 | 194 | 200 | 157 | 159 | 153 | 165 | 170 | 174 | 284 | 288 | 140 | 144 |
| BRBC | LM | 172 | 184 | 227 | 236 | 155 | 182 | 161 | 161 | X | X | 194 | 194 | 157 | 157 | 153 | 165 | 174 | 174 | 284 | 284 | 140 | 144 |
| BRS | LM | 184 | 184 | 236 | 236 | 182 | 182 | 149 | 161 | 150 | 154 | 194 | 200 | 157 | 159 | 153 | 155 | 174 | 174 | 284 | 288 | 128 | 140 |
| RGBC | LM | 184 | 184 | 232 | 236 | 155 | 167 | 161 | 161 | 150 | 154 | 194 | 194 | 157 | 159 | 155 | 165 | 174 | 174 | 284 | 284 | 144 | 144 |
| SRBC | LM | 184 | 184 | 240 | 240 | 155 | 159 | 165 | 173 | 138 | 150 | 194 | 194 | 157 | 159 | 165 | 165 | 174 | 174 | 284 | 296 | 140 | 140 |
| SSBC | LM | 172 | 184 | 249 | 253 | 151 | 171 | 149 | 157 | 154 | 154 | 194 | 194 | 157 | 159 | 143 | 169 | 174 | 174 | 284 | 284 | 140 | 140 |
| SBLC | LM | 184 | 188 | 240 | 245 | 175 | 182 | 165 | 165 | 138 | 142 | 194 | 194 | 157 | 159 | 165 | 167 | 174 | 174 | 288 | 284 | 132 | 140 |
| SRS | LM | 184 | 184 | 227 | 236 | 151 | 182 | 149 | 161 | 146 | 154 | 194 | 194 | 157 | 159 | 153 | 169 | 174 | 174 | 284 | 284 | 140 | 140 |
| SSS | LM | 172 | 172 | 245 | 245 | 151 | 175 | 157 | 161 | 146 | 154 | 194 | 196 | 157 | 159 | 153 | 167 | 170 | 174 | 288 | 296 | 140 | 140 |
| RBLC | LM | 172 | 176 | 245 | 253 | 175 | 182 | 147 | 157 | 142 | 142 | 194 | 196 | 157 | 159 | 143 | 165 | 174 | 174 | 284 | 296 | 132 | 144 |
| CRLC | LM | 184 | 184 | 245 | 245 | 159 | 171 | 161 | 161 | 142 | 142 | 194 | 200 | 157 | 159 | 143 | 155 | 170 | 182 | 284 | 296 | 140 | 140 |
| CGLC | LM | 172 | 184 | 240 | 240 | 159 | 159 | 149 | 161 | 150 | 154 | 200 | 200 | 157 | 159 | 153 | 165 | 174 | 174 | 284 | 296 | 132 | 144 |
| CBS | LM | 176 | 176 | 236 | 245 | 159 | 182 | 161 | 165 | 142 | 150 | 194 | 196 | 157 | 157 | 155 | 155 | 174 | 174 | 284 | 307 | 144 | 144 |
| CSS | LM | 172 | 172 | 236 | 249 | 155 | 159 | 157 | 165 | 146 | 146 | 194 | 196 | 157 | 157 | 143 | 165 | 174 | 174 | 284 | 288 | 140 | 144 |
| **ID** | **Group** | **D14S306** | | **D3S1766** | | **D2S1399** | | **D7S1817** | | **D20S206** | | **D8S260** | | **D8S165** | | **D1S207** | | **D17S1290** | | **C2A** | | **D5S1457** | |
| FREB | LM | 172 | 184 | 245 | 249 | 171 | 182 | 149 | 149 | 154 | 154 | 200 | 200 | 159 | 159 | 155 | 165 | 170 | 170 | 284 | 288 | 140 | 144 |
| FSRE | LM | 182 | 182 | 240 | 245 | X | X | 165 | 165 | 142 | 150 | X | X | 159 | 159 | 165 | 165 | 174 | 174 | 284 | 284 | 144 | 144 |
| FWM | LM | 176 | 184 | 253 | 253 | 171 | 182 | X | X | 138 | 150 | 194 | 194 | 157 | 159 | 165 | 171 | 174 | 174 | 284 | 288 | 140 | 144 |
| MTBM | LM | 182 | 182 | 236 | 236 | 175 | 175 | X | X | 142 | 142 | 194 | 194 | 159 | 159 | 143 | 143 | 174 | 174 | 284 | 284 | 132 | 144 |
| MTH | LM | 184 | 184 | 245 | 253 | 151 | 175 | X | X | 146 | 150 | 194 | 194 | 159 | 159 | 143 | 169 | 170 | 174 | 284 | 284 | 140 | 144 |
| SAF-TN | LM | 182 | 184 | 236 | 245 | 171 | 171 | 149 | 149 | 146 | 146 | 194 | 194 | 159 | 161 | 167 | 169 | 174 | 174 | 284 | 284 | 132 | 132 |
| SAMCUT | LM | 176 | 188 | 236 | 236 | 171 | 175 | 157 | 161 | 138 | 142 | 194 | 200 | 157 | 159 | 165 | 171 | 182 | 182 | 284 | 284 | 140 | 140 |
| TNO1 | LM | 180 | 184 | 249 | 253 | 159 | 175 | 161 | 165 | 138 | 154 | 194 | 194 | 157 | 157 | 155 | 169 | 174 | 182 | 296 | 300 | 136 | 136 |
| TNO2 | LM | 176 | 182 | 227 | 236 | 159 | 167 | 161 | 169 | 150 | 154 | 194 | 194 | 157 | 159 | 171 | 171 | 174 | 178 | 284 | 288 | 140 | 140 |
| WH | LM | 176 | 176 | 236 | 249 | 155 | 167 | X | X | 138 | 142 | 194 | 200 | 157 | 157 | 175 | 153 | X | X | 288 | 288 | 136 | 140 |
| K-30_A | K30 | 172 | 184 | 240 | 245 | 151 | 171 | 143 | 165 | 138 | 150 | 194 | 196 | 157 | 159 | 165 | 171 | 174 | 178 | 284 | 284 | 140 | 140 |
| K-30_B | K30 | 172 | 184 | 245 | 249 | 162 | 182 | 161 | 161 | 138 | 154 | 194 | 200 | 159 | 159 | 153 | 153 | 174 | 174 | 284 | 284 | 136 | 140 |
| K-30_C | K30 | 184 | 184 | 245 | 249 | 171 | 171 | 147 | 165 | 138 | 154 | 194 | 200 | 157 | 159 | 169 | 169 | 174 | 178 | 284 | 284 | 140 | 144 |
| K-30_D | K30 | 176 | 180 | 232 | 245 | 171 | 182 | 149 | 165 | 142 | 154 | 194 | 196 | 157 | 157 | 153 | 165 | 174 | 174 | 284 | 308 | 140 | 144 |
| K-30_E | K30 | 172 | 184 | 240 | 245 | 155 | 171 | X | X | 138 | 150 | 194 | 196 | 157 | 159 | 165 | 171 | 170 | 174 | 284 | 284 | 140 | 140 |
| DURA_A | DUR | 172 | 176 | 232 | 249 | 167 | 171 | 147 | 147 | 150 | 154 | 194 | 200 | 157 | 157 | 153 | 167 | 174 | 174 | 284 | 288 | 132 | 136 |
| DURA_B | DUR | 172 | 176 | 232 | 249 | 167 | 171 | 149 | 161 | 150 | 154 | 194 | 194 | 157 | 159 | 143 | 163 | 170 | 174 | 284 | 296 | 132 | 136 |
| DURA_C | DUR | 172 | 180 | 232 | 236 | 175 | 182 | 149 | 165 | 138 | 146 | 194 | 200 | 157 | 161 | 165 | 171 | 174 | 174 | 284 | 288 | 136 | 140 |
| DURA_D | DUR | 172 | 180 | 208 | 245 | 159 | 171 | 161 | 161 | 138 | 154 | 194 | 194 | 157 | 157 | 155 | 165 | 170 | 178 | 288 | 296 | 132 | 132 |
| DURA_E | DUR | 172 | 180 | 232 | 236 | 175 | 182 | 161 | 165 | 138 | 146 | 194 | 194 | 157 | 159 | 165 | 165 | 170 | 174 | 284 | 284 | 140 | 140 |
| SEB_A | SEB | 176 | 184 | 236 | 249 | 162 | 167 | 157 | 147 | 142 | 150 | 196 | 200 | 157 | 157 | 143 | 143 | X | X | 307 | 307 | X | X |
| SEB_B | SEB | 180 | 184 | 227 | 232 | 151 | 159 | 161 | 161 | 146 | 146 | 194 | 196 | 159 | 159 | 165 | 165 | 170 | 170 | 288 | 296 | 132 | 140 |
| SEB_C | SEB | 180 | 184 | 227 | 232 | 151 | 159 | 161 | 165 | 146 | 146 | 194 | 194 | 159 | 159 | 165 | 165 | 170 | 178 | 288 | 296 | 140 | 144 |
| SEB_D | SEB | 172 | 184 | 240 | 249 | 159 | 175 | 173 | 143 | 142 | 150 | 194 | 194 | 157 | 157 | 153 | 165 | 174 | 174 | 284 | 284 | 136 | 144 |
| SEB_E | SEB | 172 | 184 | 240 | 253 | 155 | 167 | 143 | 165 | 138 | 150 | 194 | 200 | 157 | 159 | 143 | 165 | 174 | 178 | 284 | 284 | 140 | 144 |
| SEB_F | SEB | 172 | 184 | X | X | 159 | 171 | 157 | 161 | 138 | 142 | 194 | 200 | 157 | 157 | 143 | 155 | 174 | 174 | 288 | 288 | 140 | 140 |
| MAIN_A | MAIN | 176 | 184 | 208 | 232 | 155 | 159 | 177 | 177 | 138 | 146 | 194 | 194 | 157 | 159 | 161 | 161 | 174 | 178 | 288 | 288 | X | X |
| MAIN_B | MAIN | 176 | 188 | 204 | 204 | 175 | 186 | 157 | 161 | 129 | 162 | X | X | 161 | 161 | 155 | 155 | 174 | 178 | 252 | 252 | 124 | 130 |
| MAIN_C | MAIN | 184 | 188 | 245 | 245 | 159 | 171 | 149 | 165 | 146 | 150 | 194 | 196 | 157 | 159 | 143 | 171 | 170 | 174 | 284 | 288 | 128 | 136 |
| MAIN_D | MAIN | 172 | 188 | 240 | 245 | 155 | 159 | 147 | 157 | 142 | 150 | 194 | 200 | 157 | 157 | 169 | 173 | 170 | 174 | 284 | 296 | 144 | 144 |
| MAIN_E | MAIN | 176 | 184 | 208 | 232 | 155 | 159 | 157 | 177 | 138 | 146 | 194 | 194 | 157 | 159 | 143 | 155 | 174 | 174 | 284 | 288 | 144 | 144 |
