# Supplementary material for: Primate DNA suggests long-term stability of an African rainforest
Source: Ecol Evol. 2012 Oct 9;2(11):2829–42. doi: 10.1002/ece3.395 (PMC3501634; doi:10.1002/ece3.395)

**APPENDIX B**

The Maximum Clade Credibility Trees for each of the 10 loci for two models of molecular evolution, (I) our best fit model – PU2 and (II) EU1the model that most approximates a stepwise mutation model. The time scale is in terms of years.

***Part I. Model PU2.***

***Figure S3:*** Locus C2A


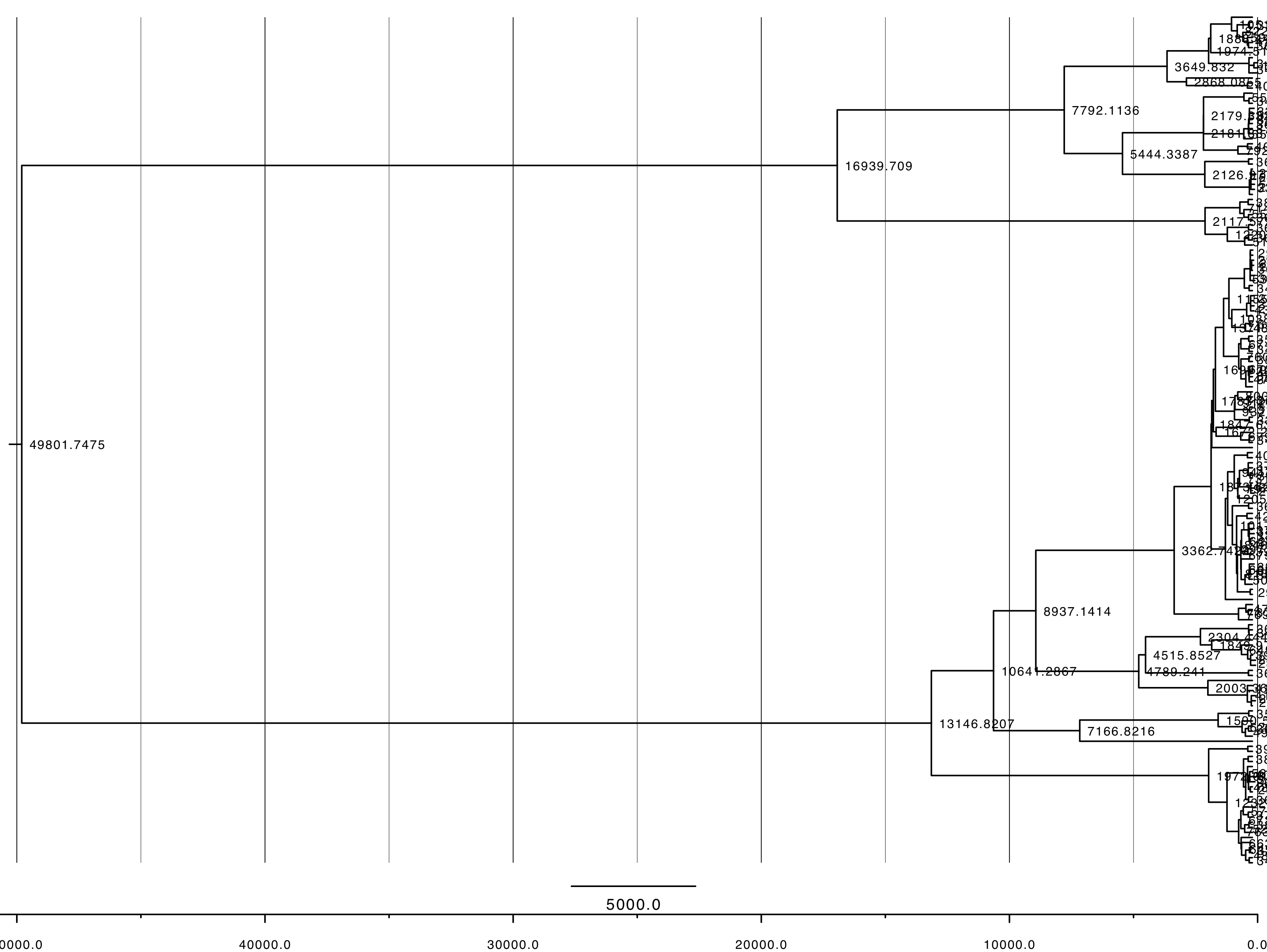


***Figure S4:*** Locus D1S207


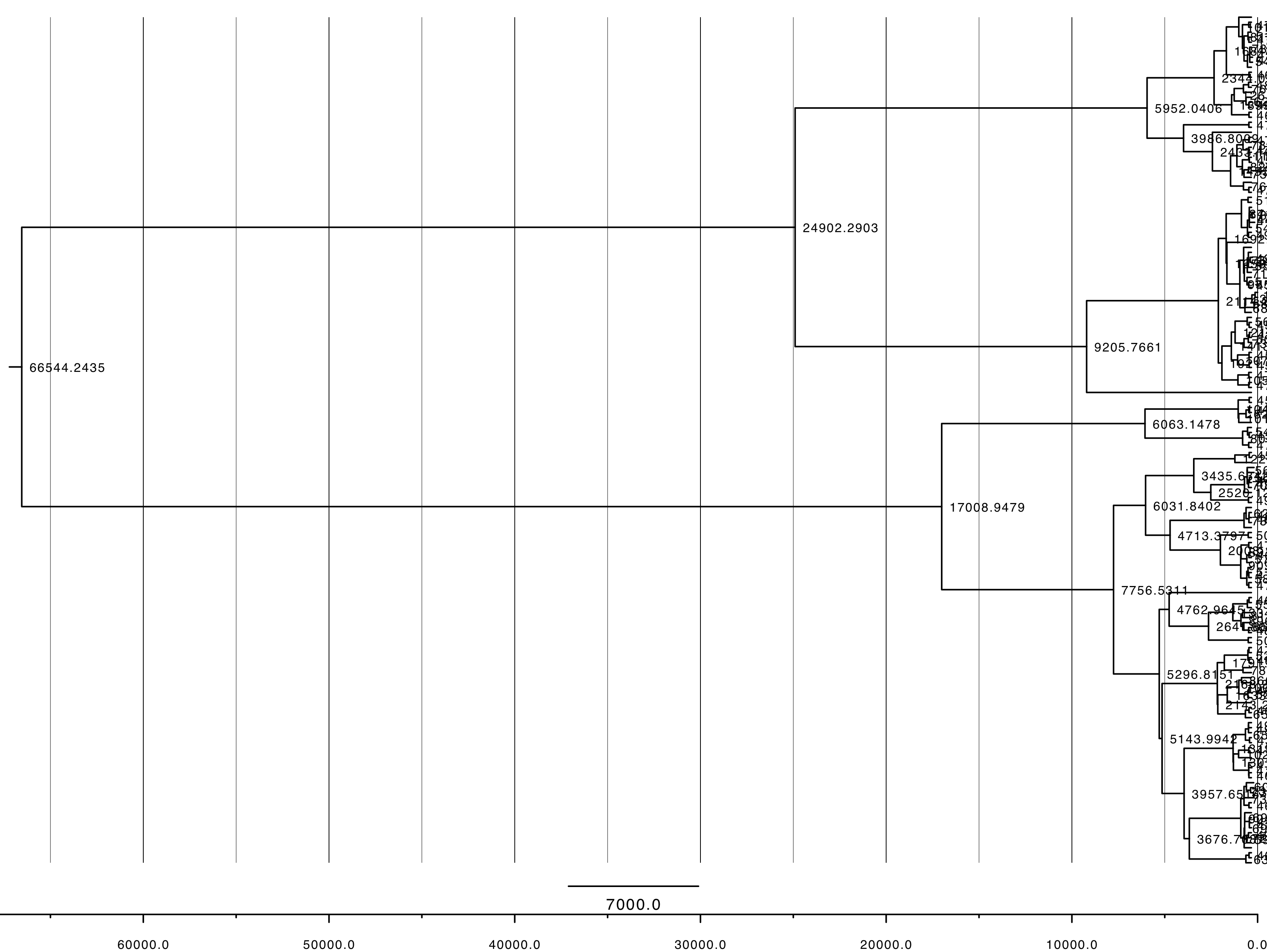


***Figure S5:*** Locus D2S1399


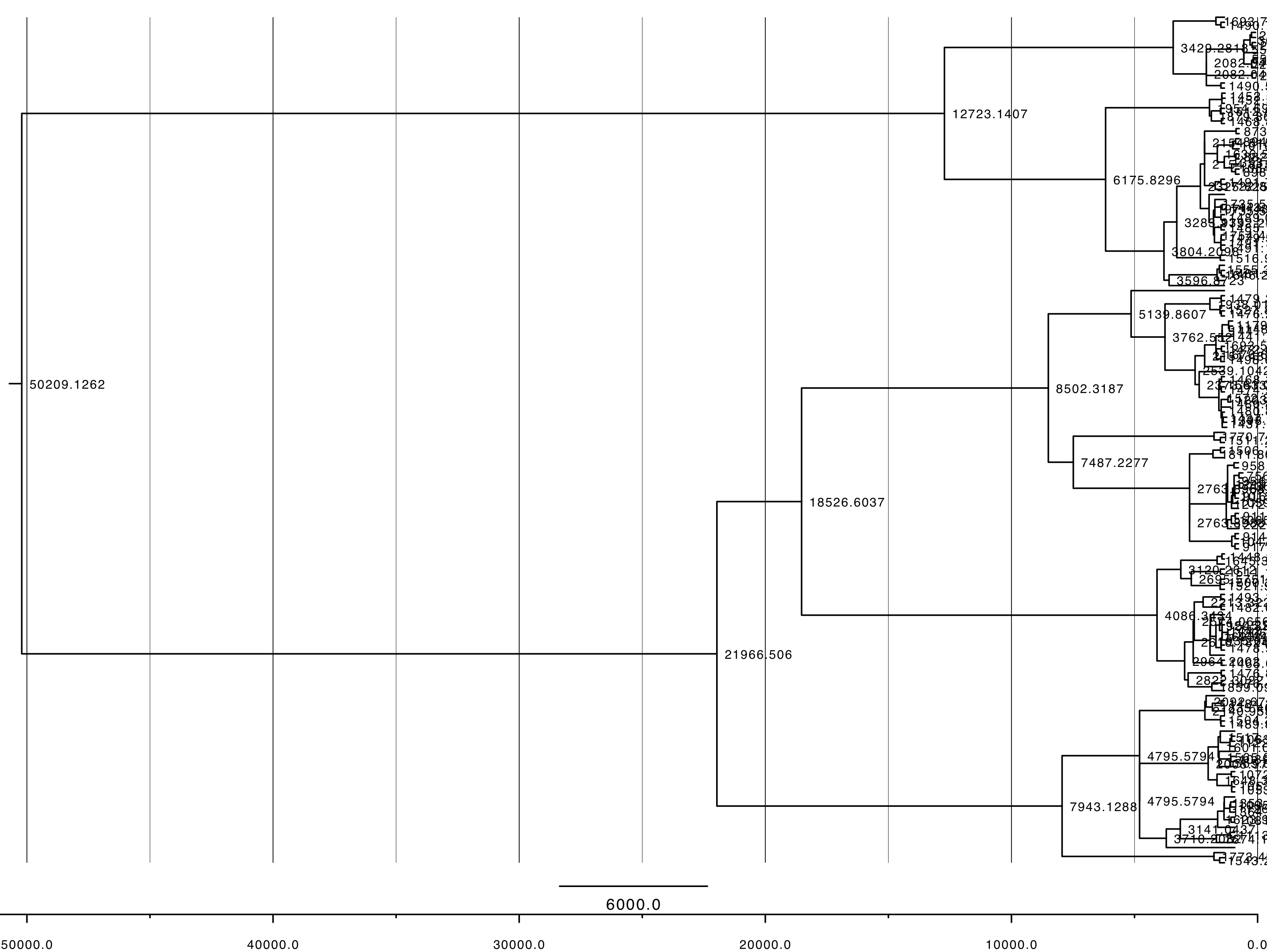


***Figure S6:*** Locus D3S1766


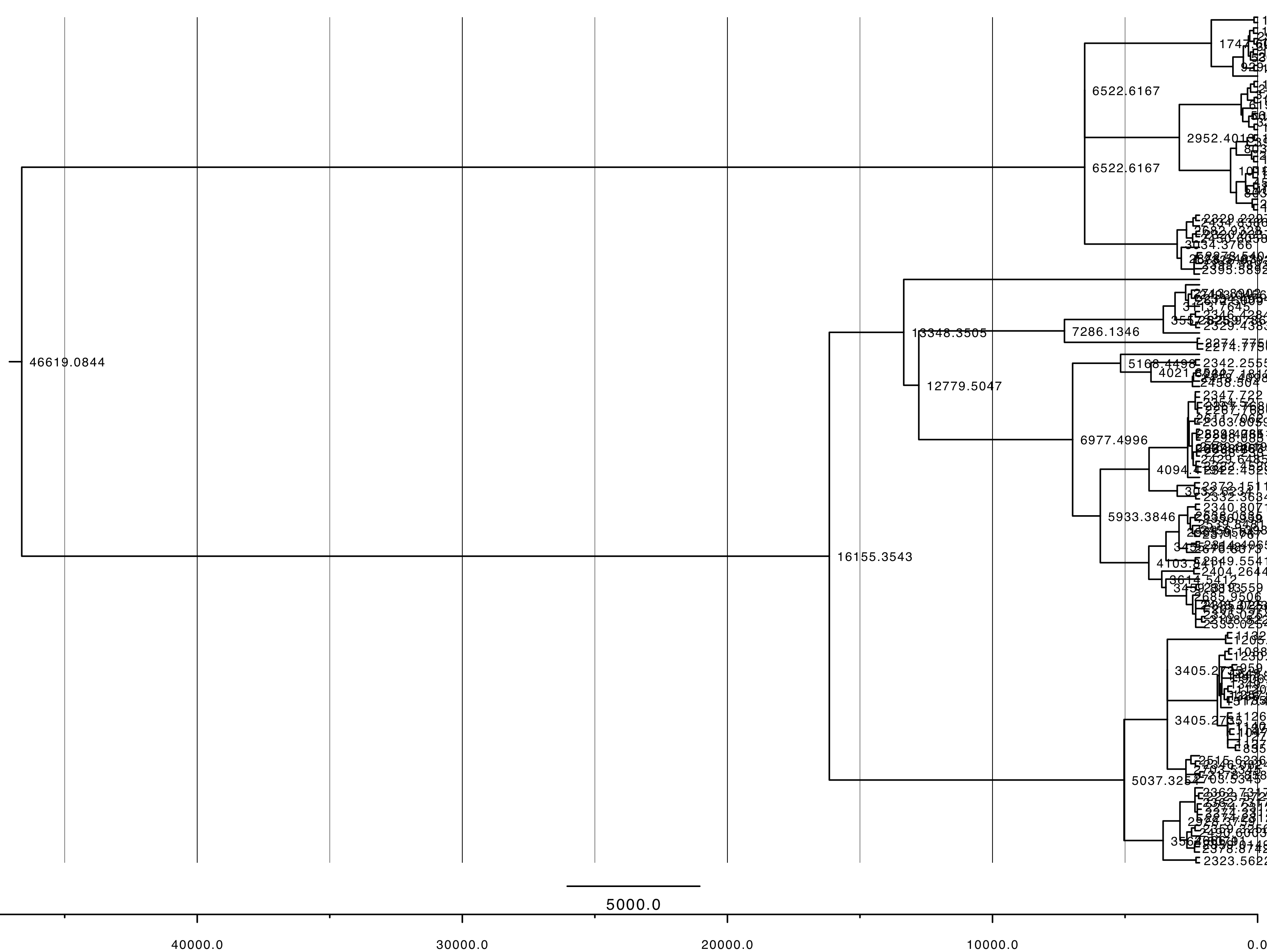


***Figure S7:*** *Locus* D5S1457


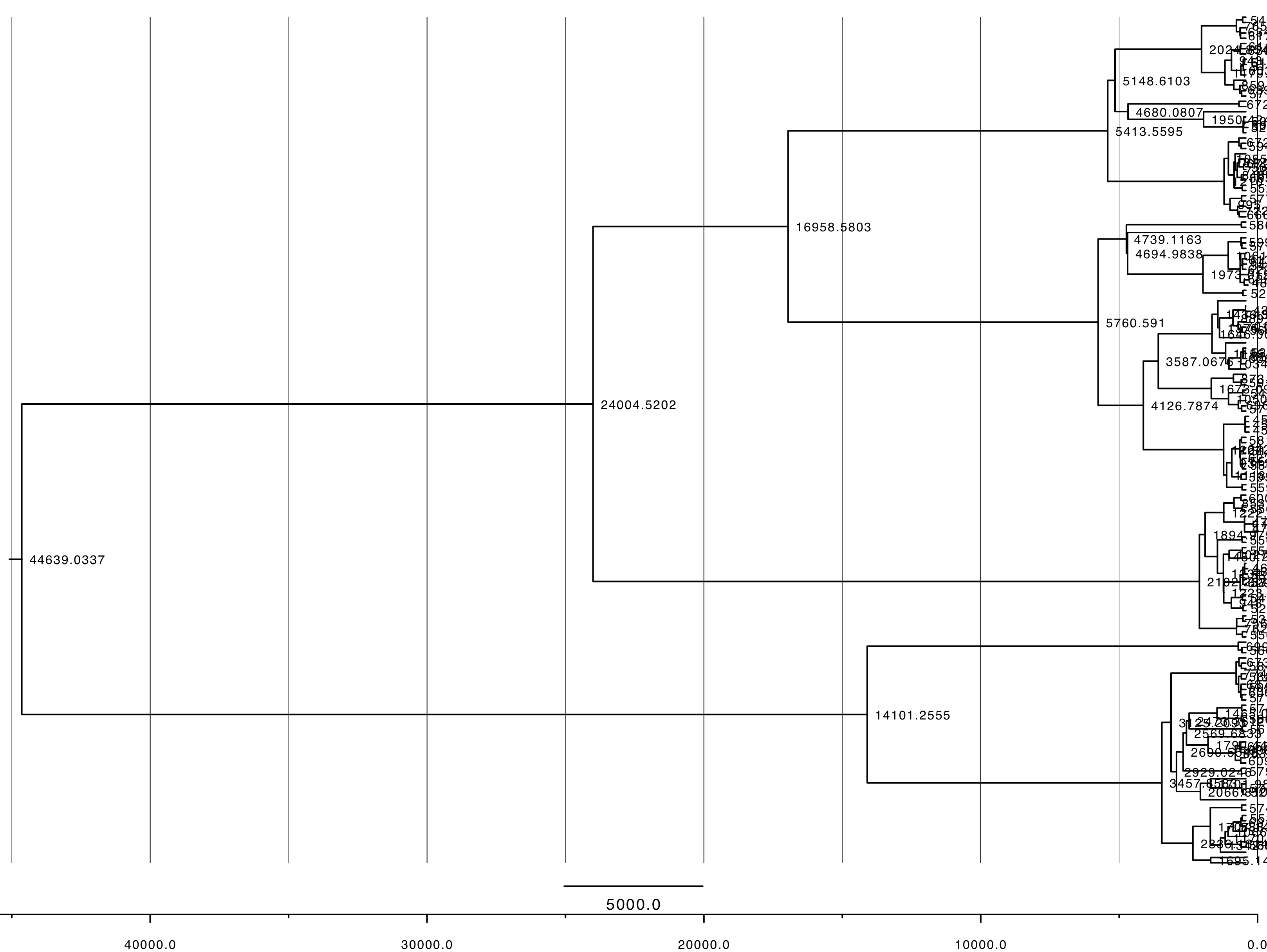


***Figure S8:*** Locus D7S1817


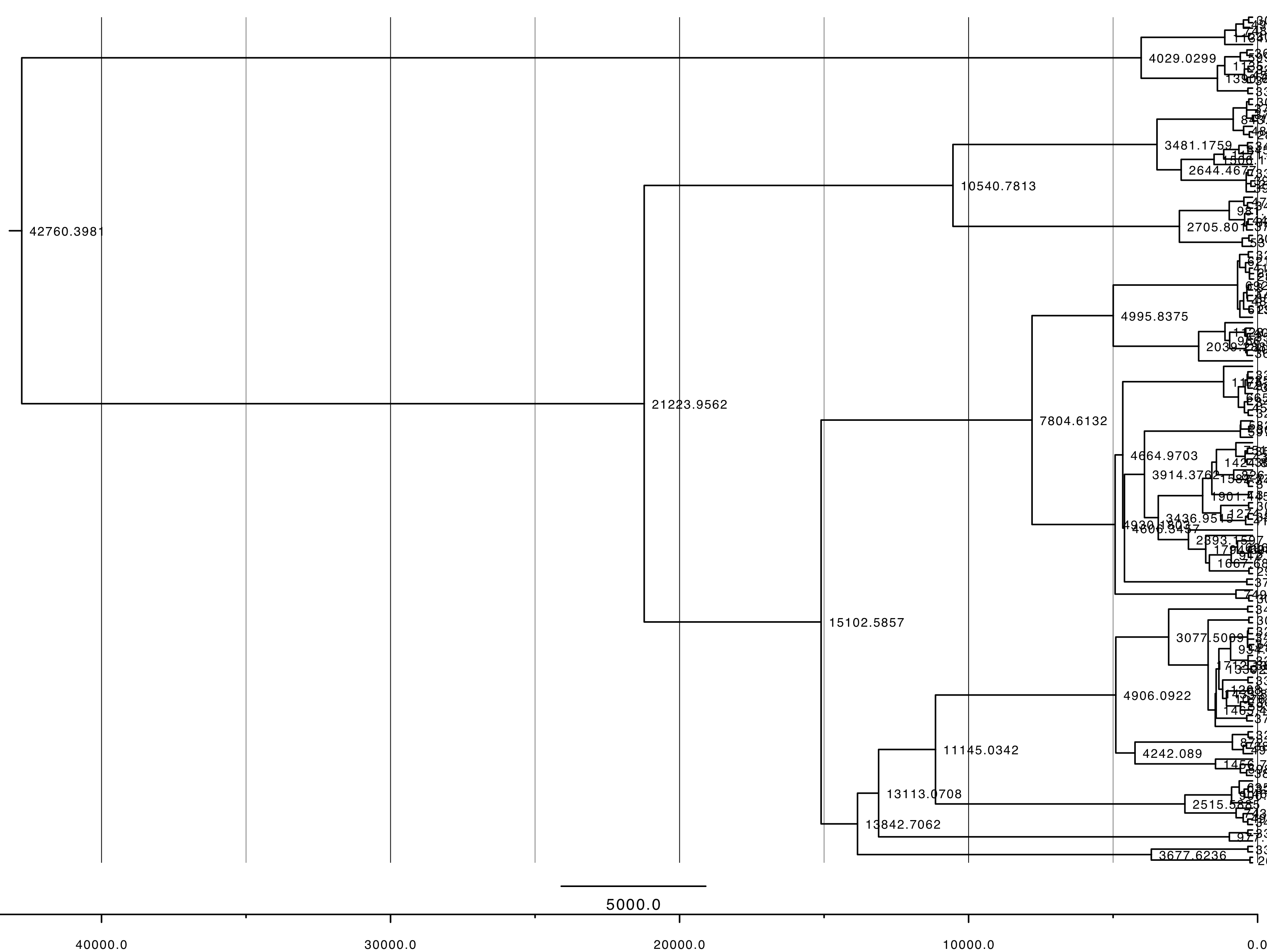


***Figure S9:*** Locus D8S165


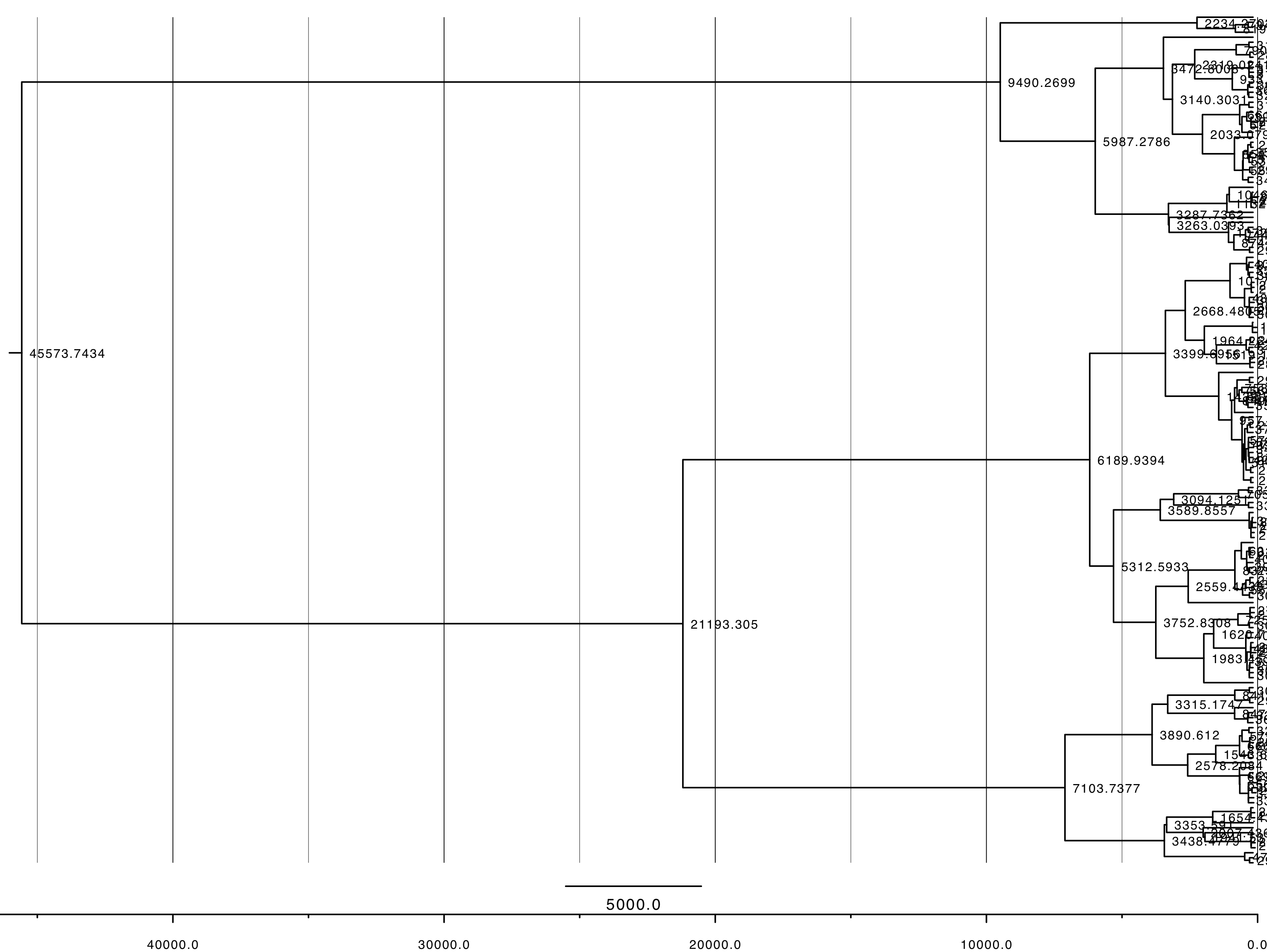


***Figure S10:*** Locus D8S260


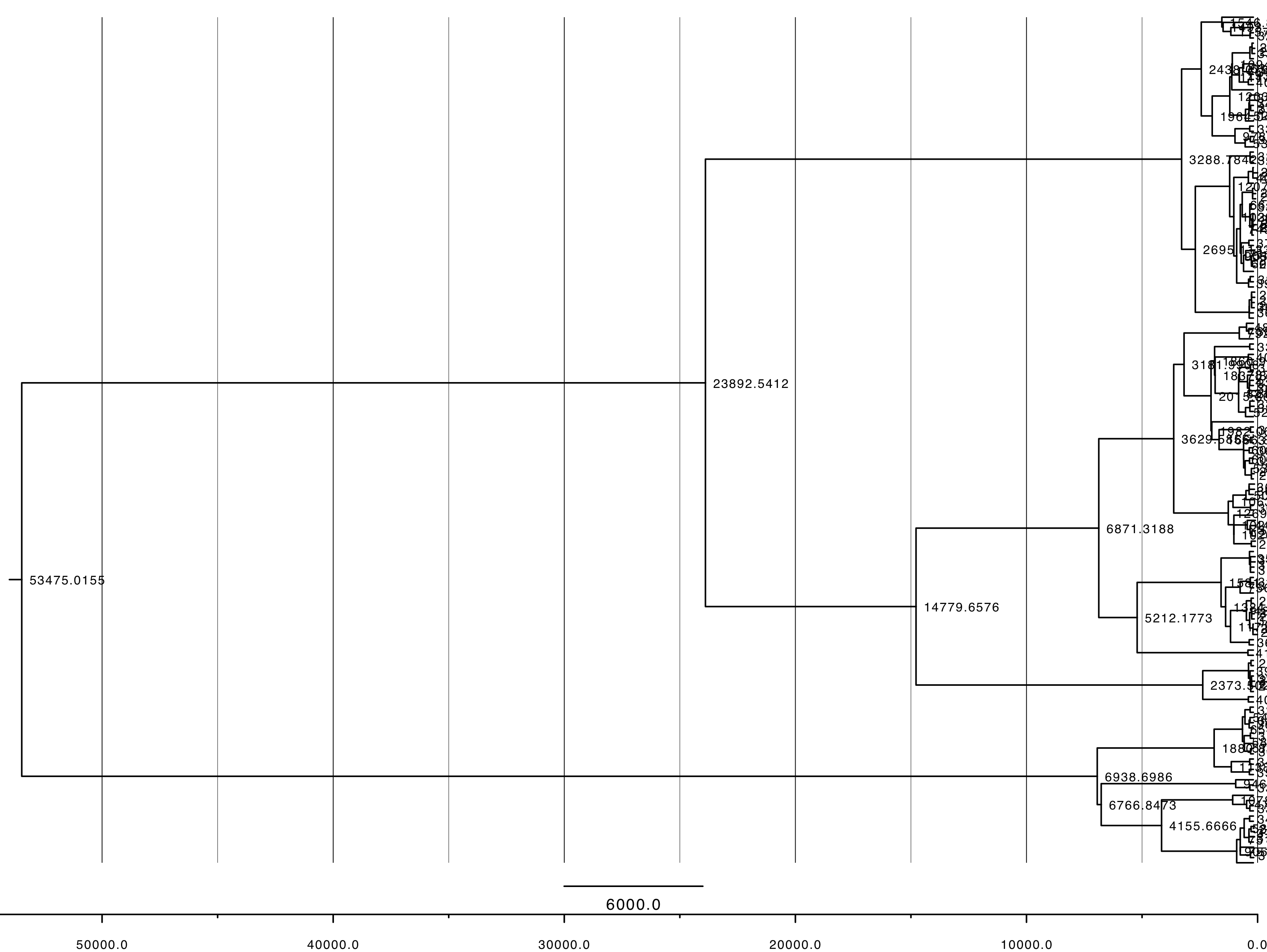


***Figure S11:*** Locus D14S306


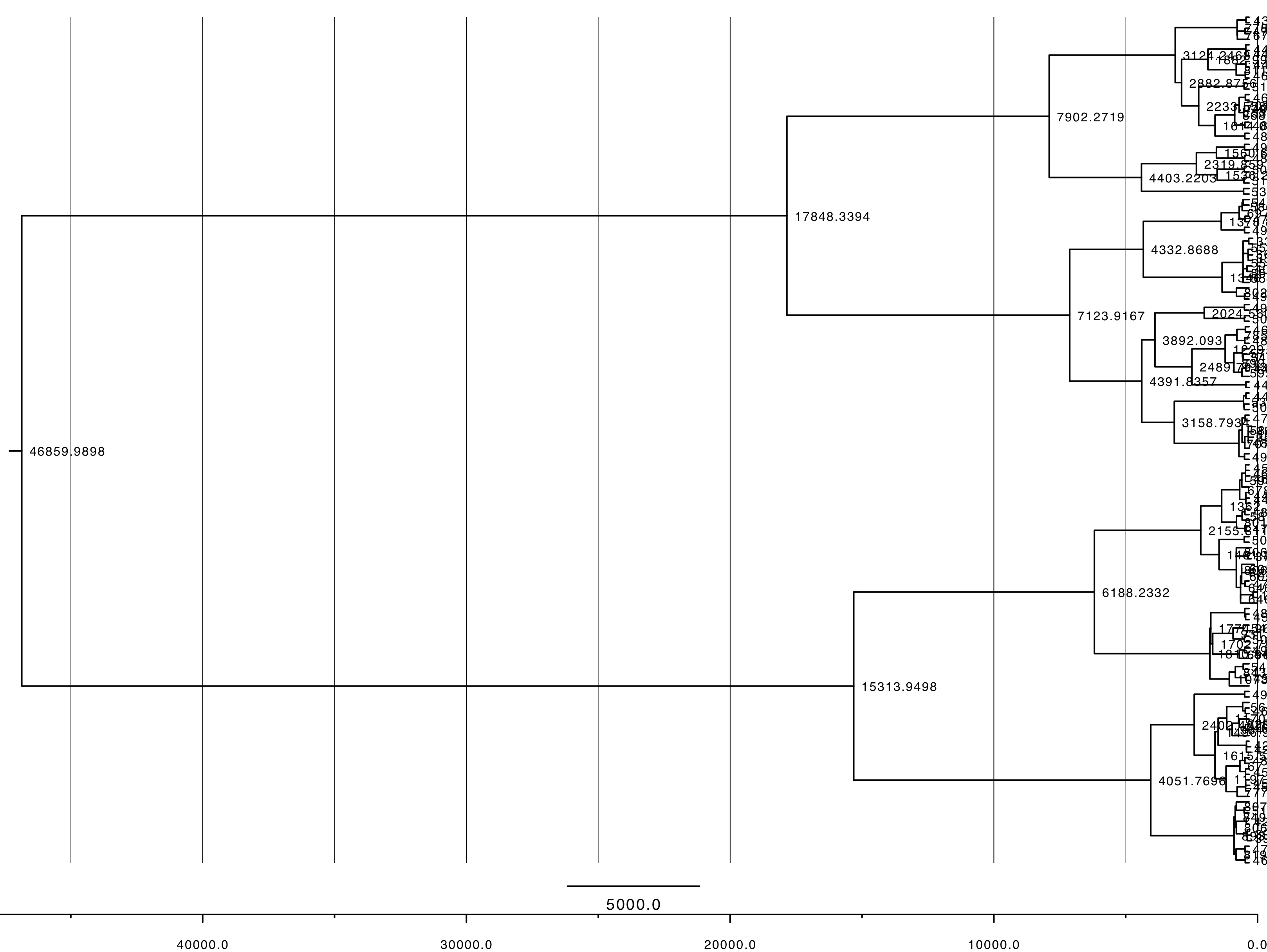


***Figure S12:*** Locus D20S206


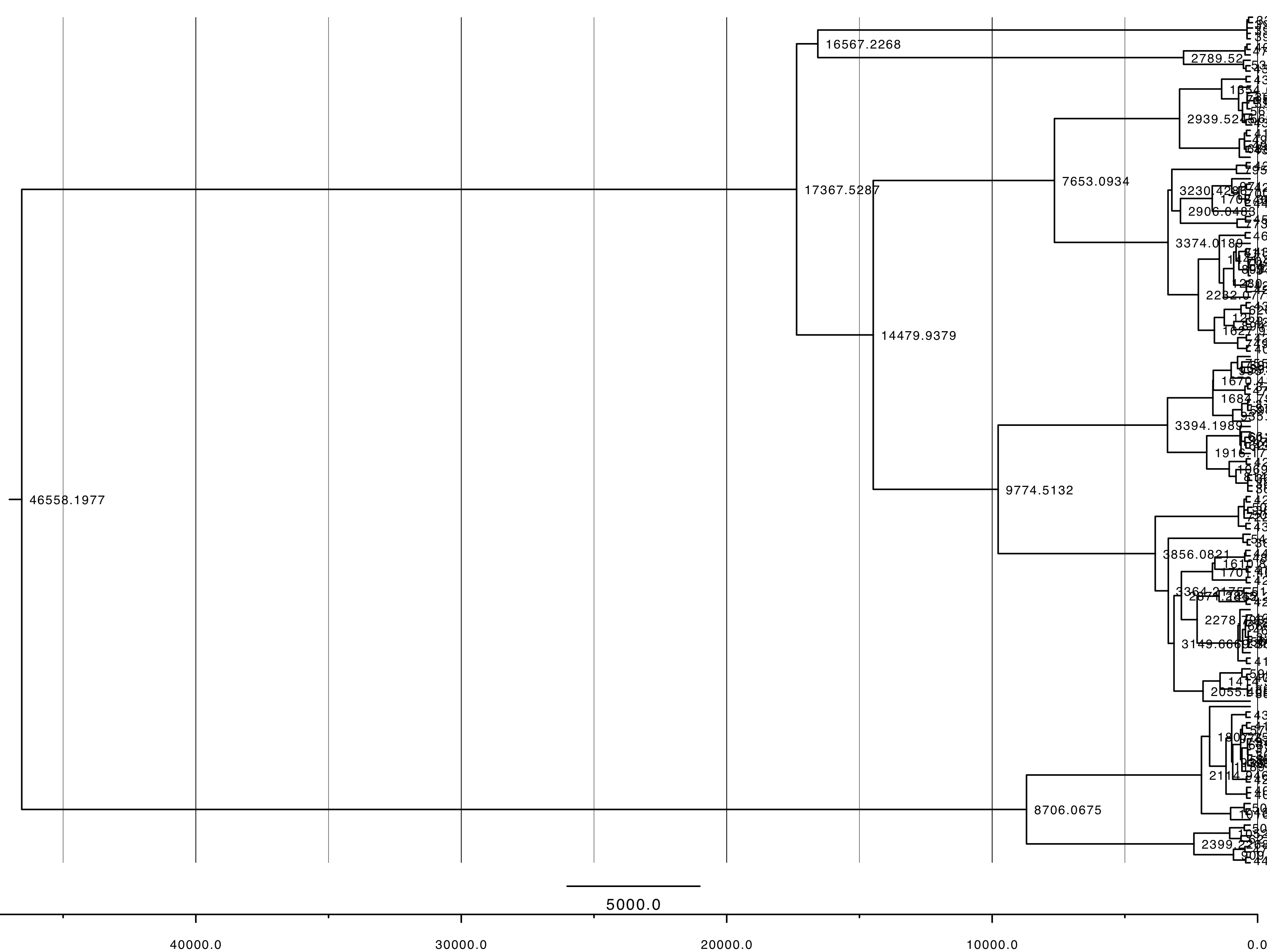


***Part II. Model EU1.***

***Figure S13:*** Locus C2A


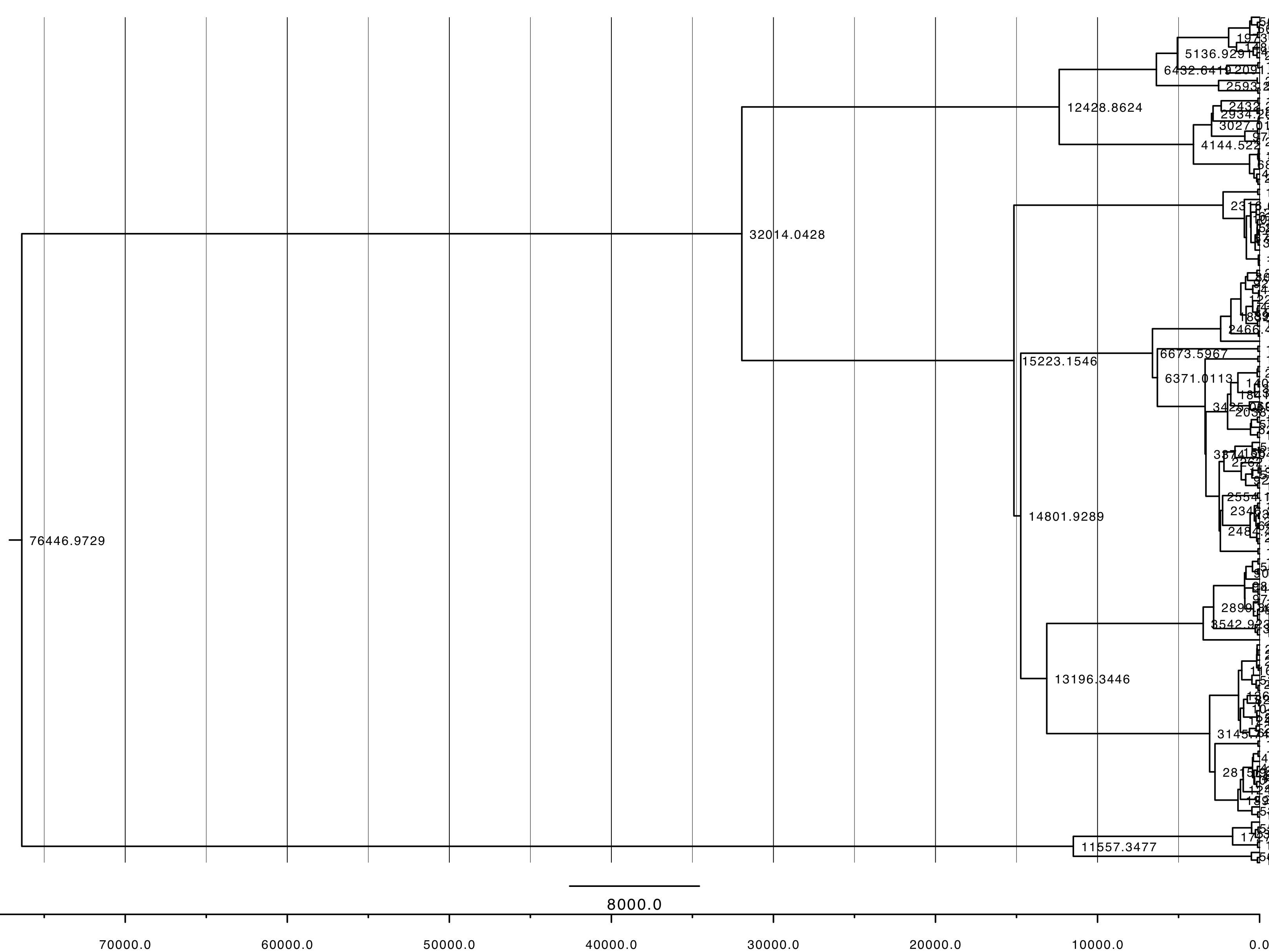


***Figure S14:*** Locus D1S207


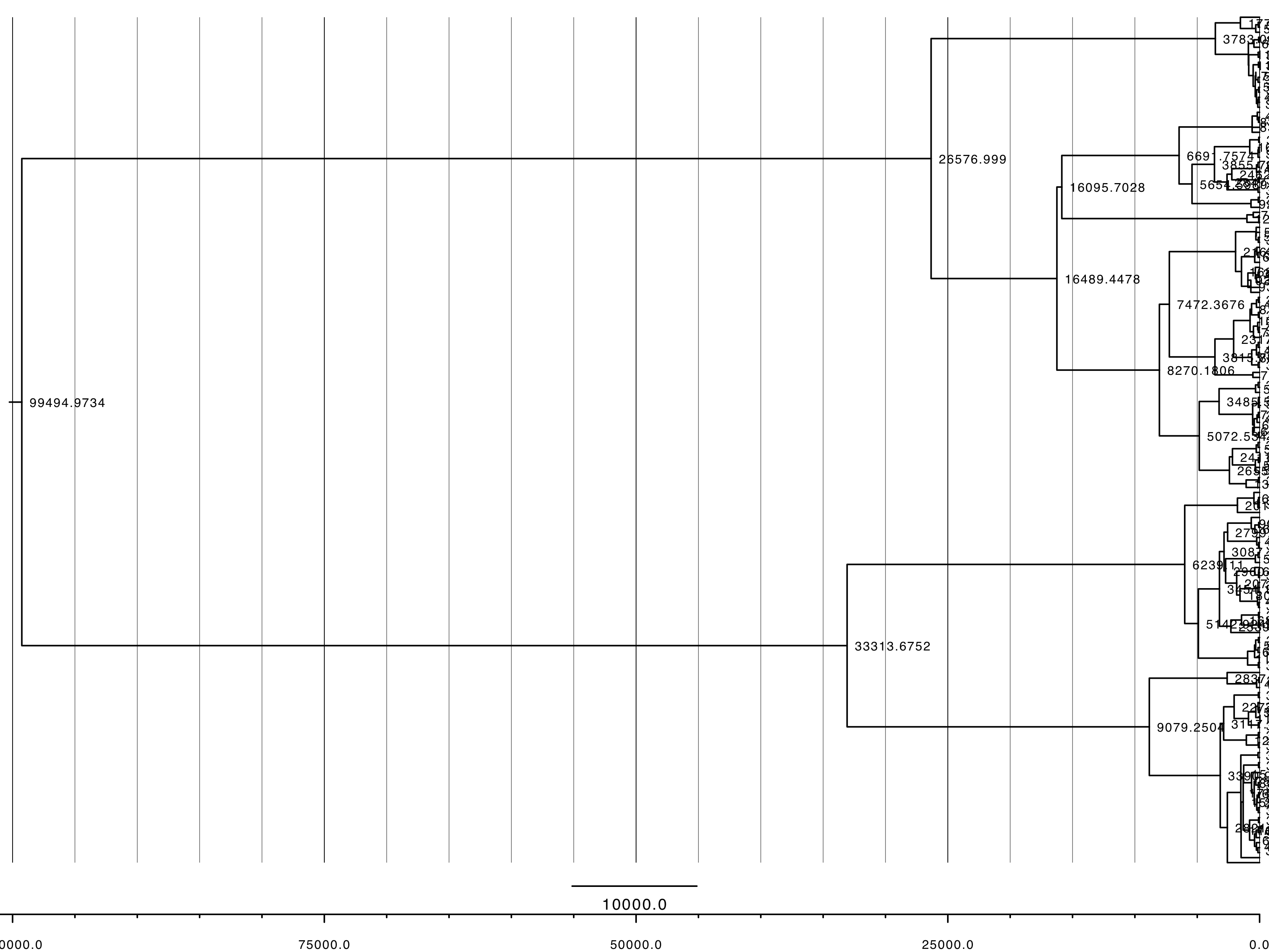


***Figure S15:*** Locus D2S1399


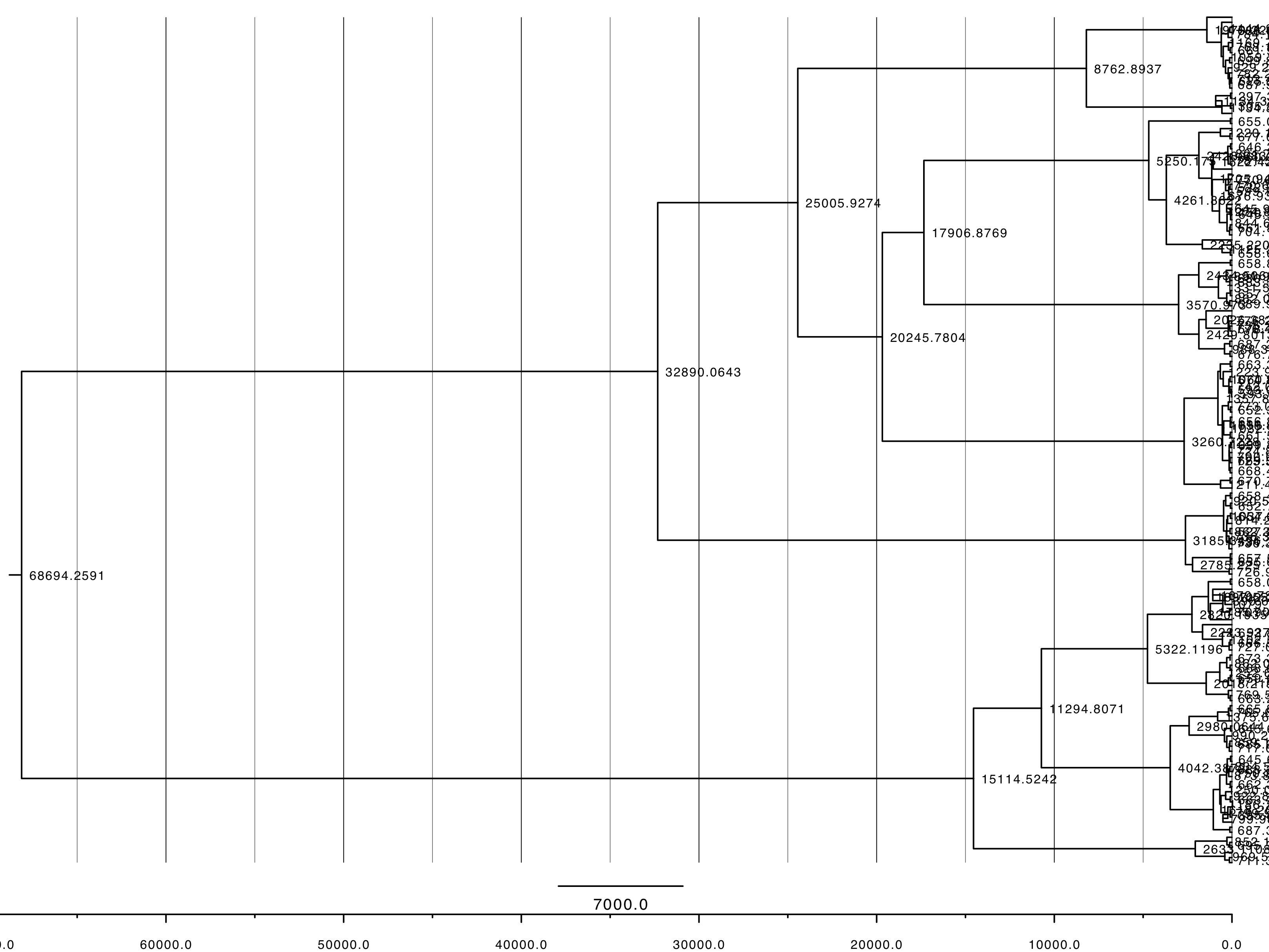


***Figure S16:*** Locus D3S1766


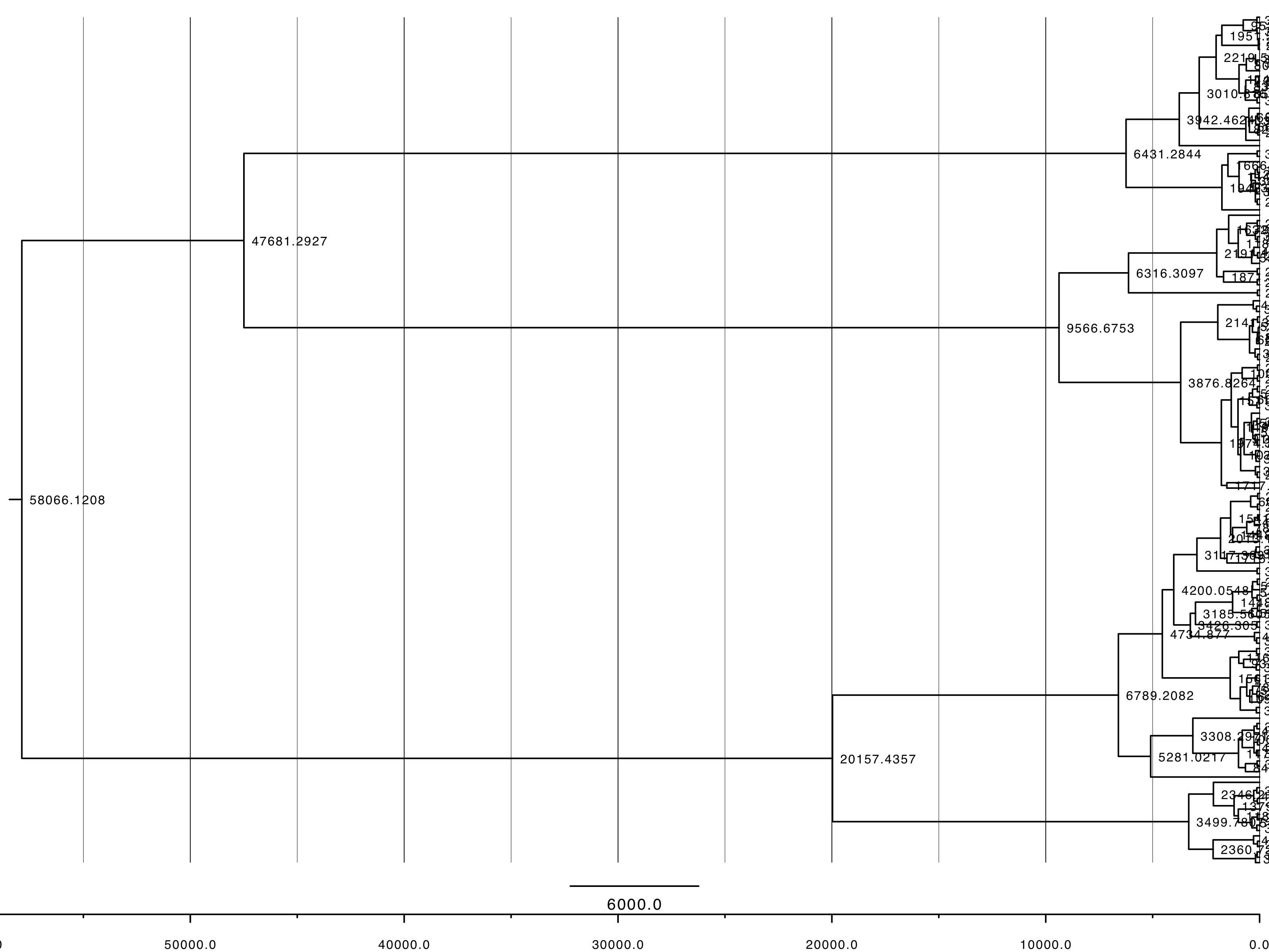


***Figure S17:*** Locus D5S1457


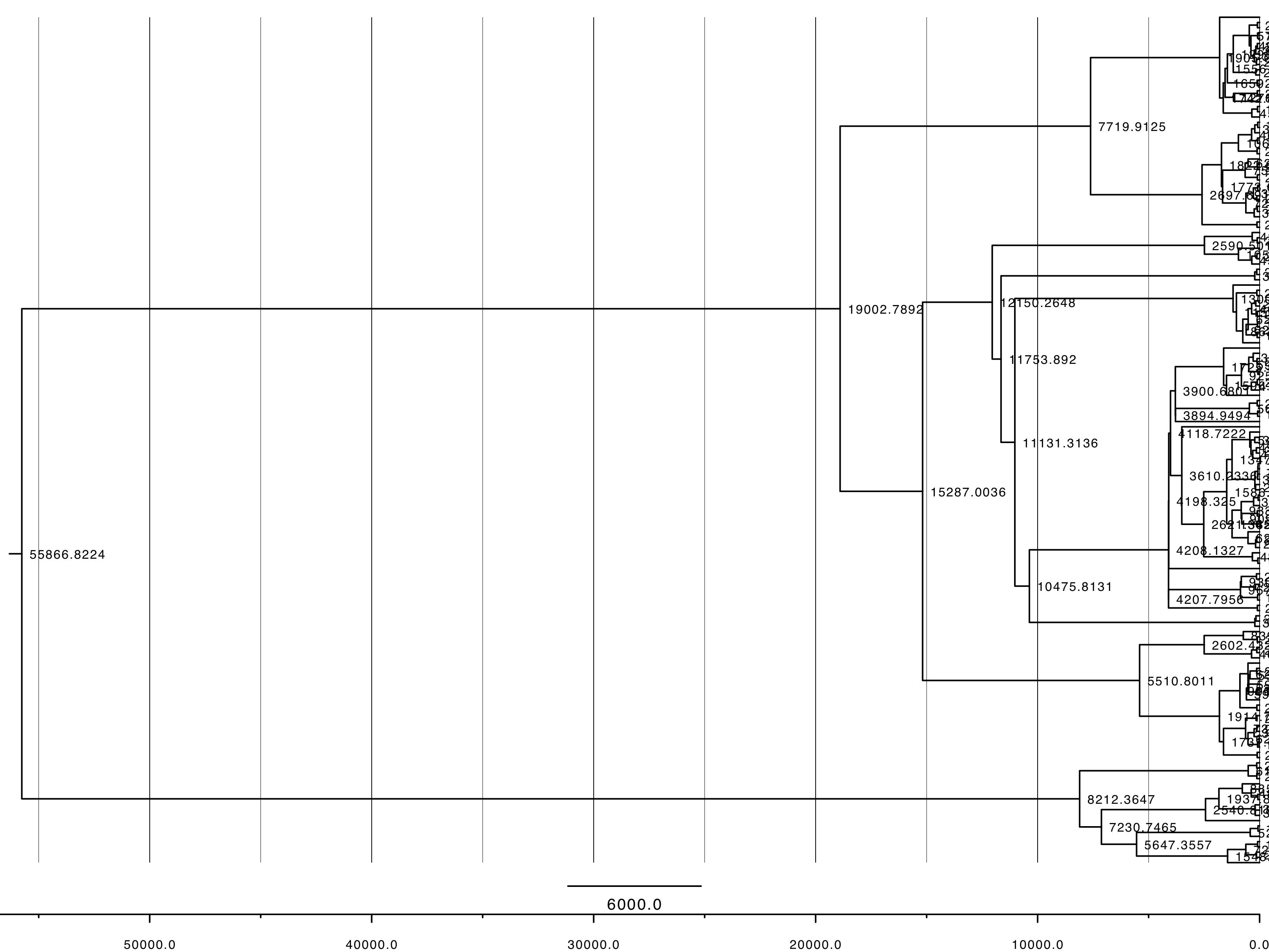


***Figure S18:*** Locus D7S1817


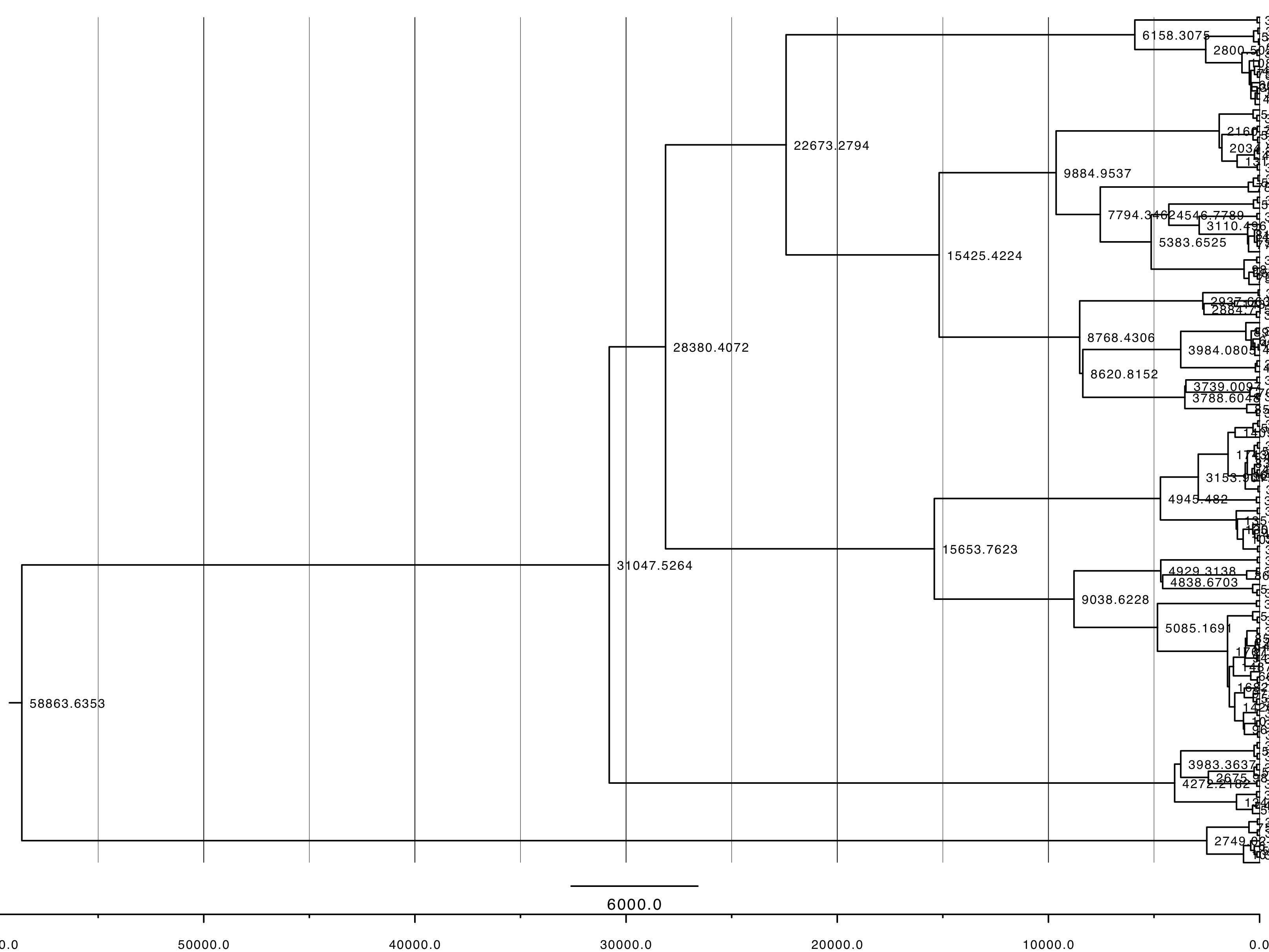


***Figure S19:*** Locus D8S165


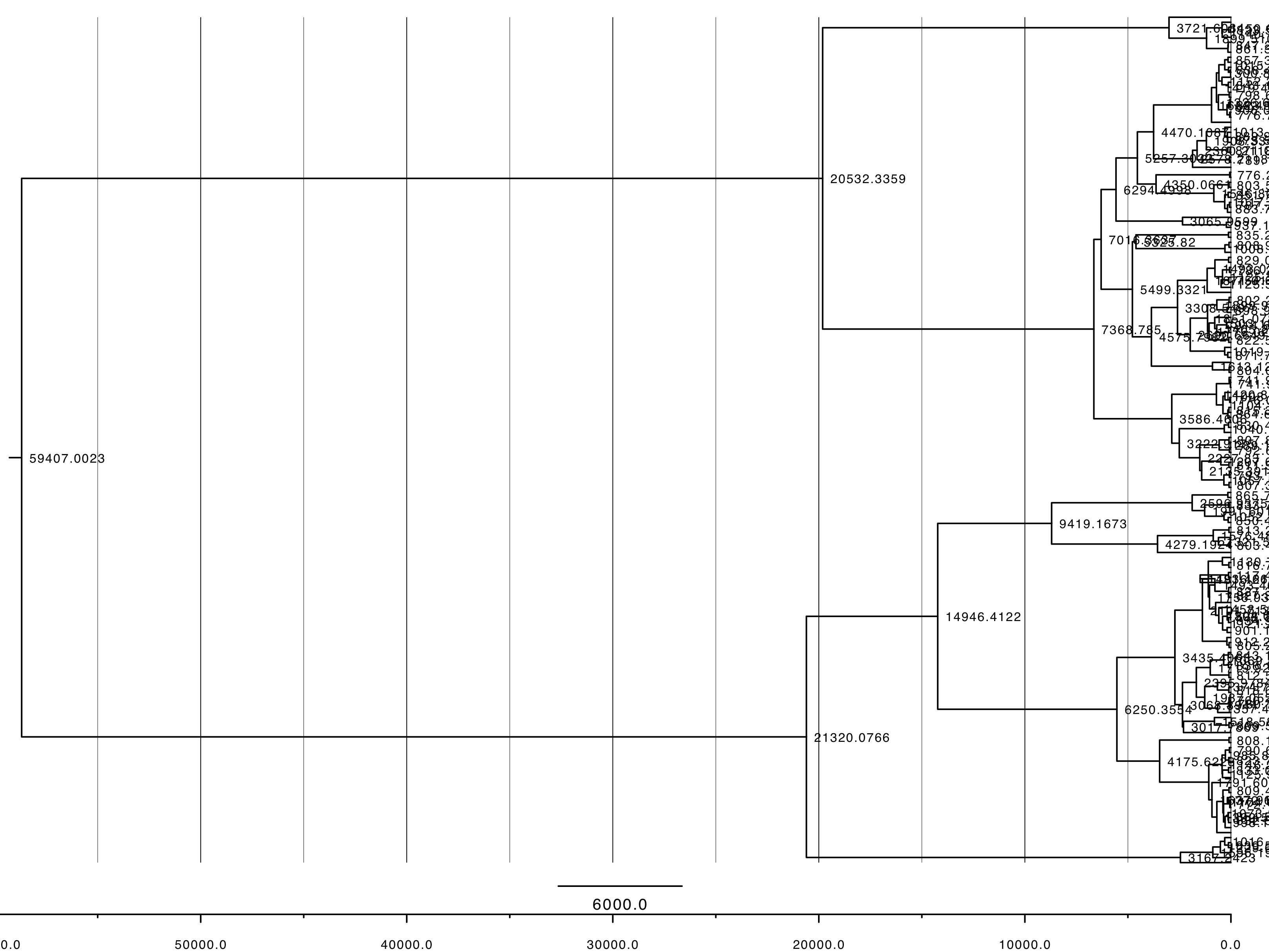


***Figure S20:*** Locus D8S260


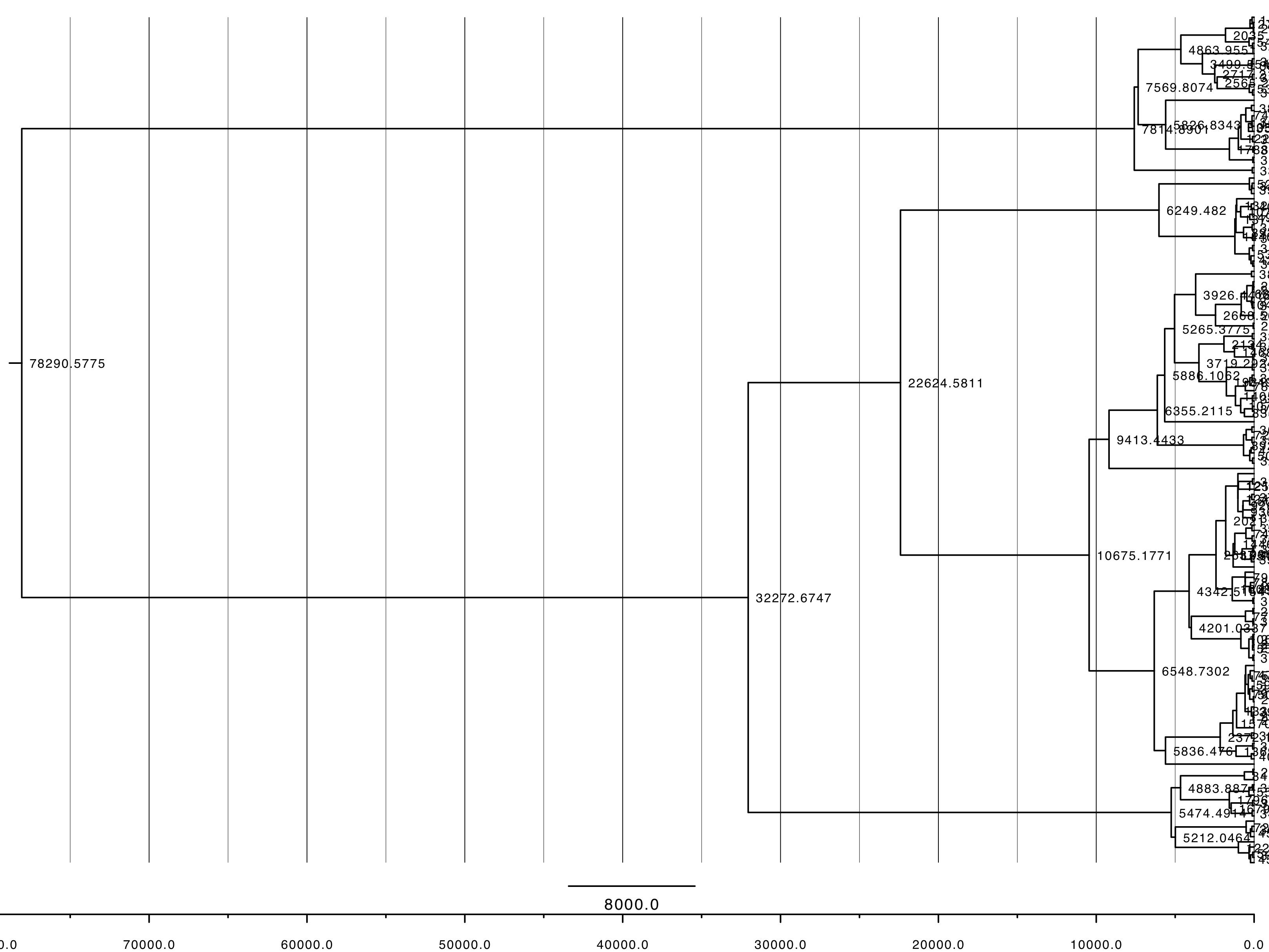


***Figure S21:*** Locus D14S306


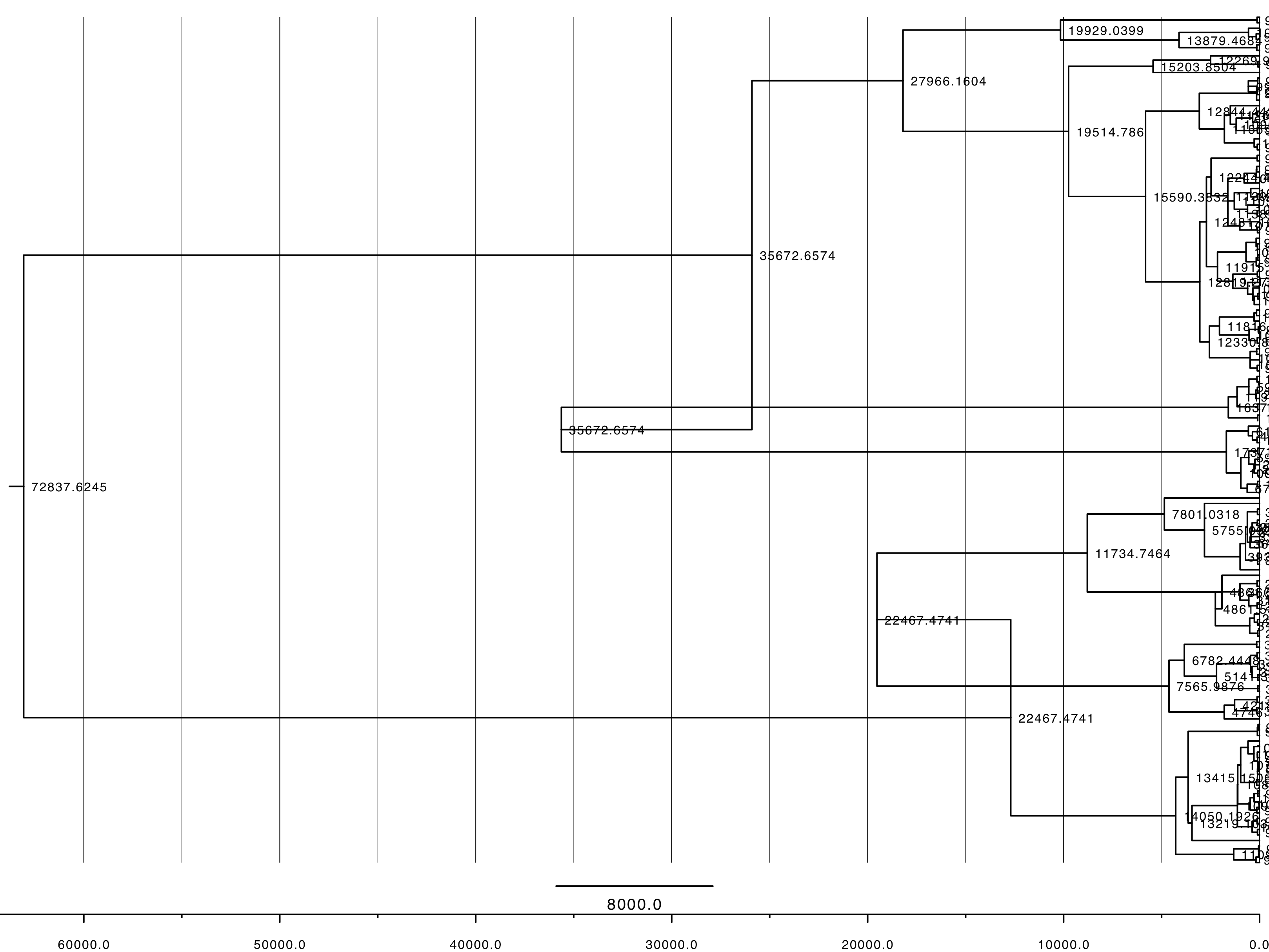


***Figure S22:*** Locus D20S206


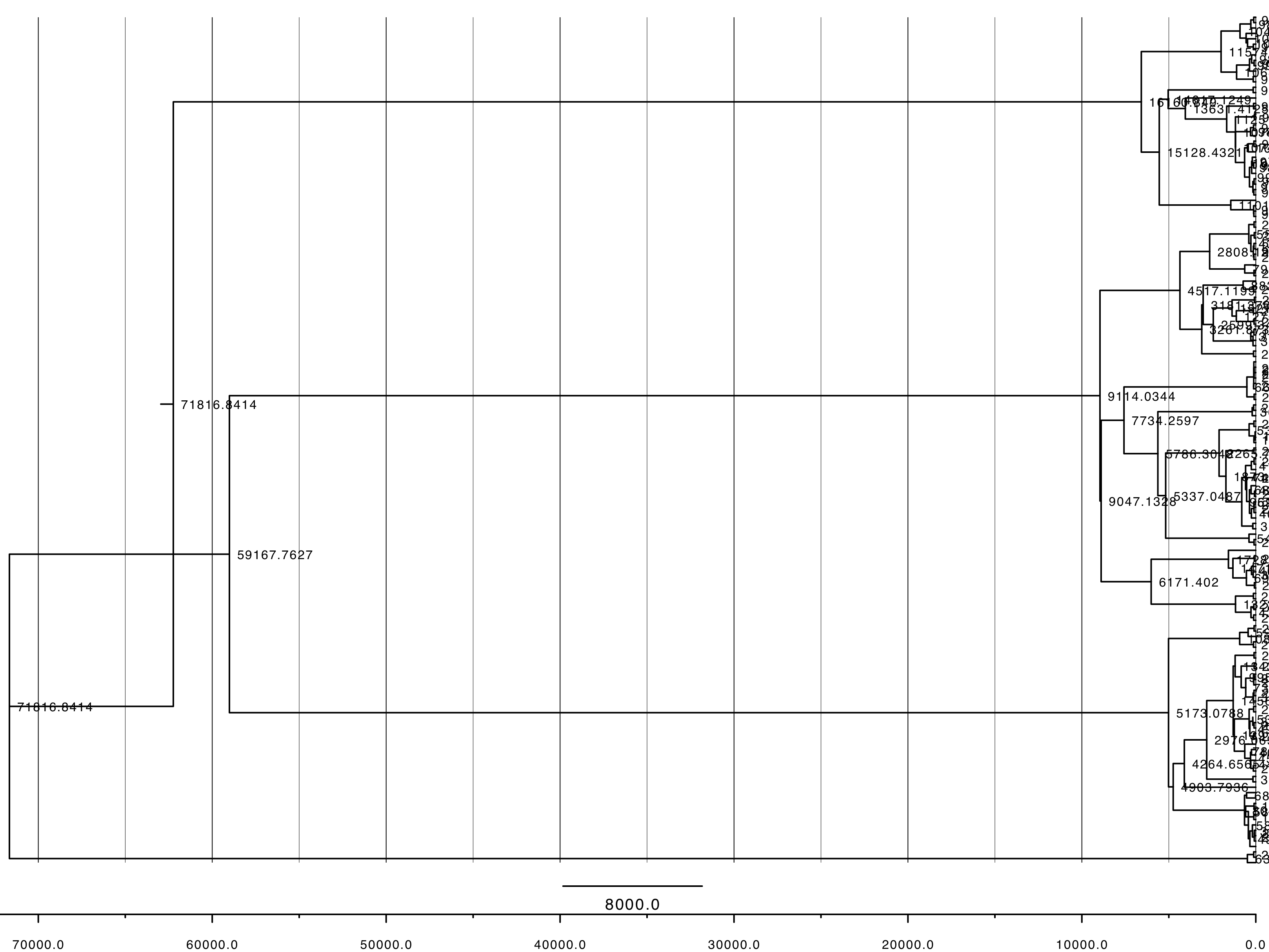

Supplement: Supplementary file 2 [file ece30002-2829-SD2.doc]
